# DNA methylation patterns in peripheral blood mononuclear cells from Holstein cattle with variable milk yield

**DOI:** 10.1186/s12864-018-5124-9

**Published:** 2018-10-11

**Authors:** Chad D Dechow, Wan-Sheng Liu

**Affiliations:** Department of Animal Science, College of Agricultural Sciences, The Pennsylvania State University, 324 Henning Building, University Park, State College, PA 16802 USA

**Keywords:** Epigenetics, Selection, *Bos taurus*, MeDIP-seq, DNA methylation

## Abstract

**Background:**

Milk yield for Holstein cows has doubled over five decades due to genetic selection and changes to management, but the molecular mechanisms that facilitated this increase are mostly unknown. Epigenetic modifications to the cattle genome are a plausible molecular mechanism to cause variation in milk yield and our objective was to describe genome-wide DNA methylation patterns in peripheral blood mononuclear cells (PBMC) from mature Holstein dairy cows with variable milk yield.

**Results:**

Whole genome MeDIP-seq was performed following DNA extraction from PBMC of 6 lactating dairy cows from 4 different herds that varied in milk yield from 13,556 kg to 23,105 kg per 305 day lactation. We describe methylation across the genome and for 13,677 protein coding genes. Repetitive element reads were primarily mapped to satellite (36.4%), SINE (29.1%), and LINE (23.7%) regions and the majority (78.4%) of CpG sites were sequenced at least once. DNA methylation was generally low upstream of genes with the nadir occurring 95 bp prior to the transcription start site (TSS). Methylation was lower in the first exon than in later exons, was highest for introns near the intron-exon junctions, and declined downstream as the distance from the gene increased. We identified 72 differentially methylated regions (DMR) between high milk yield cows and their control, and 252 DMR across herd environments.

**Conclusions:**

This reference methylome for cattle with extreme variation in milk yield phenotype provides a resource to more fully evaluate relationships between DNA methylation and phenotype in populations subject to selection. The detection of DMR in cows of varying milk yield suggests potential to exploit epigenetic variation in cattle improvement programs.

**Electronic supplementary material:**

The online version of this article (10.1186/s12864-018-5124-9) contains supplementary material, which is available to authorized users.

## Background

Molecular mechanisms that confer phenotypic diversity and that facilitate selective change include DNA methylation [1]. Indeed, genetic selection may act partly through altered epigenetic profiles as DNA sequence variation is reported to cause shifts in DNA methylation [2].

Holstein (*Bos taurus*) cattle are the most numerous of dairy cattle breeds and produce the largest volumes of milk, fat and protein [3]. Selection for yield in Holstein cattle has increased genetic merit for milk production by 3713 kg, a ~ 59% increase, since 1960 [4]. Changes to cow housing, feeding, and management have increased milk yield by an additional 2503 kg, with the combined effect of genetic selection and management resulting in an approximate doubling of milk yield in five decades. While highly successful, the genes and physiological processes which have been altered to facilitate such increases remain elusive. A notable exception is a binucleotide substitution in the *diacylglycerol O-acyltransferase 1* (*DGAT1*) gene that causes a lysine to alanine substitution at position 232 (*K232A*) [5, 6]. The alanine variant results in higher milk and protein yield, but is not economically advantageous in many markets because of a substantial correlated decline in milk-fat yield. *DGAT1* is the largest quantitative trait locus (QTL) for milk, fat, and protein yield [7] reported to date.

More recently, with the development and application of the Illumina BovineSNP50 BeadChip [8] and subsequent BeadChips of varying marker density [9], dairy cattle selection programs have incorporated genomic predictions facilitated by marker genotypes for thousands of loci spread across the genome [10]. Genomic analysis has largely confirmed the quantitative model of many small effects that cumulatively account for a high degree of variation [11], but understanding of how selection alters performance remains elusive. Collaborative efforts to sequence a large reference population of cattle from many breeds, such as the 1000 bull genomes project [12], will provide further insights into DNA sequence variation and potential impacts of such variation on cattle phenotypes [13], but increasing gene marker density beyond ~ 50,000 markers has not improved the accuracy of genomic predictions by a substantial degree to date [14].

While significant efforts have been expended studying associations of DNA sequence variation with cattle performance, epigenetic variations and their contribution to milk yield and cow health have received little attention and there are no known epigenetic-QTL for milk yield. Of particular interest to dairy cattle breeders would be linkages between epigenetic variation and the health of cows. Selection for yield [15], higher levels of confinement, and shifts in housing and feeding stratagies [16] are unfavorably associated with cow health and wellbeing. Emerging evidence from humans has shown a role for epigenetics in important cattle diseases [17] such as metabolic failure [18, 19], respiratory infection [20], and lymphoma [21], but this has not been studied extensively in livestock species.

Shifts in DNA methylation are plausible molecular mechanisms for phenotypic change in milk yield and cow health due to selection and management [22], but the cattle methylome must be more completely described before such effects can be determined [23]. The objective of this study was to identify genome-wide DNA methylation patterns in Holstein peripheral blood mononuclear cells (PBMC) to provide a resource for further investigation into causes of phenotypic variation in high milk-yield dairy cows.

## Results

### Animals

Blood was collected from the coccygeal vein (tail vein) of 6 lactating Holstein dairy cows from four commercial Pennsylvania dairy farms. Results from their official genomic evaluation for milk, fat and protein yields plus phenotypic records for yield during the parity of DNA sampling are reported in Table 1.

**Table 1:** Official genomic estimated breeding values, observed yields, age, and parity of DNA sampling

Cow	Description	gEBV Milk (kg)	gEBV Fat (kg)	gEBV Protein (kg)	Milk (kg)	Fat (kg)	Protein (kg)	Age (mo.)	Parity
F1H	Farm 1 Case	624	8	5	23,105	1028	674	61	2
F1L	Farm 1 Control	− 1316	−26	−25	15,798	498	477	71	2
F2H	Farm 2 Case	− 967	−12	−34	19,000	764	533	84	4
F2L	Farm 2 Control	− 740	−56	−34	13,556	454	369	85	5
F3I	Farm 3	− 907	−57	−40	16,094	576	443	69	4
F4I	Farm 4	171	−15	−6	16,379	685	463	116	4

gEBV = genomic estimated breeding values provided from the Council on Dairy Cattle Breeding; positive values indicate that cows are expected to produce more milk, fat or protein than the average cow born in 2010 and negative values indicate cows expected to produce less. Milk, fat and protein yields (kg) are standardized to a constant 305 day parity length and age [54]

Four cows represented case-control pairs and the protocol for selecting these pairs is described in the methods. All cows were housed in tie-stalls and fed a total-mixed ration that was top-dressed according to the cow’s nutritional requirements. The intermediate milk yield cows were from farms that allowed pasture access for lactating cows during the summer, whereas the case-control cows remained confined during lactation. The high milk yield cows averaged 21,052 kg of milk during the parity of blood sampling, whereas control cows averaged 14,677 kg of milk. The 2 remaining cows were selected from separate herds to increase the number of cows and environments represented and had milk yield that was intermediate (16,236 kg) to the high and control cows. Identifying cows with extremely high milk yield required that we sample cows from generally well-managed herds; consequently, our control cows had milk yield that was somewhat higher than the national average of 12,087 kg for Holsteins [3].

### General methylation patterns in the genome of Holstein cows

**Fig. 1:**
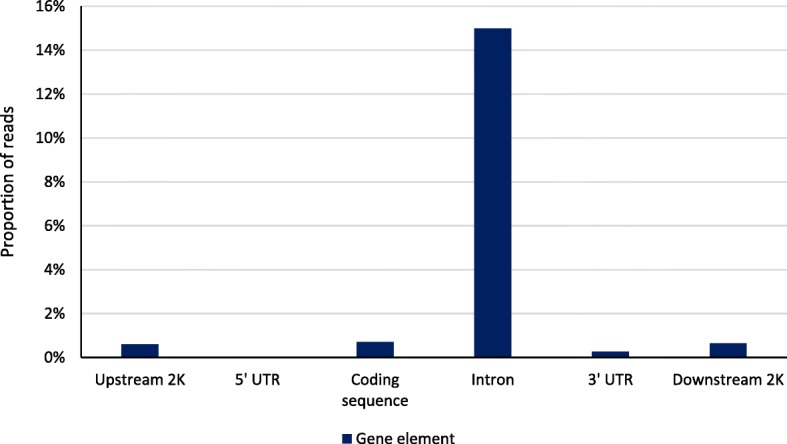
Proportion of reads mapped to gene elements. Percent of reads mapped 2 kb upstream of the transcription start site, in the 5′-untranslated region, in the protein coding sequence, in introns, in the 3′-untranslated region, and 2 kb downstream of the transcription termination site

**Fig. 2:**
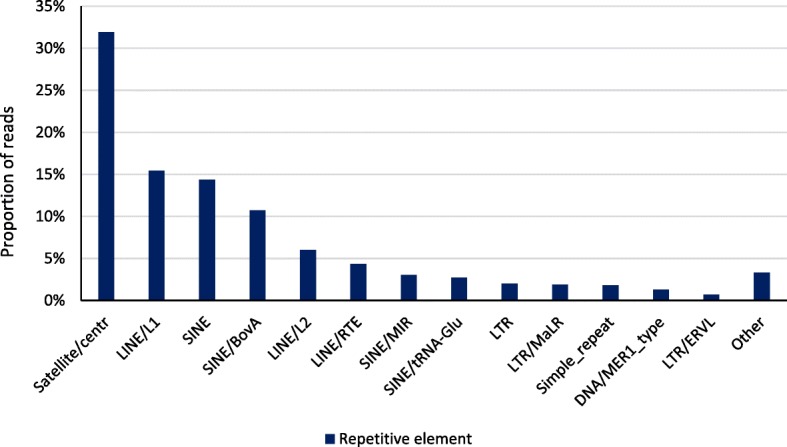
Proportion of repetitive element reads mapped to each different element. Percent of repetitive element reads mapped to satellite/centromeric, LINE, SINE, long terminal repeats, and other types of repetitive DNA sequence

**Fig. 3:**
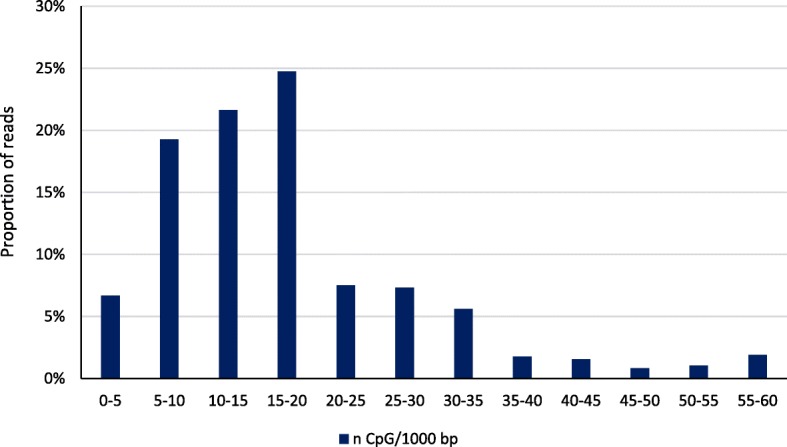
Proportion of reads in relationship to the number of CpG sites per kb. The percentage of reads mapped to genomic regions by the relative density of CpG sites per kb

Clean reads were aligned to btau4.0 with an average across cows of 64% of clean reads uniquely mapped; of these 60% were mapped to repetitive elements and 40% to unique sequences. The proportion of unique reads mapped to non-repetitive elements near gene bodies is shown in Fig. 1. These reads were most likely to be mapped to introns (15.0%); the combined proportion for the other gene elements totaled 2.2% with the 5′-untranslated region accounting for only 0.03% of reads. Figure 2 reports the proportion of repetitive element reads, which were primarily mapped to satellite (36.4%), SINE (29.1%), and LINE (23.7%) regions. The majority (78.4%) of CpG sites were sequenced at least once and 19.8% were sequenced at a depth of 5 or greater. In contrast, the genome-wide sequencing depths were 28.5% and 3.7% for depths of ≥1 or ≥ 5, respectively. CHG (H = A, C, or T) sites (43.5%) were more likely to be sequenced at least once than CHH sites (38.5%). The proportion of reads mapped by CpG density is shown in Fig. 3. Approximately two-thirds of reads were mapped to regions that contained between 5 and 20 CpG per kb.

**Fig. 4:**
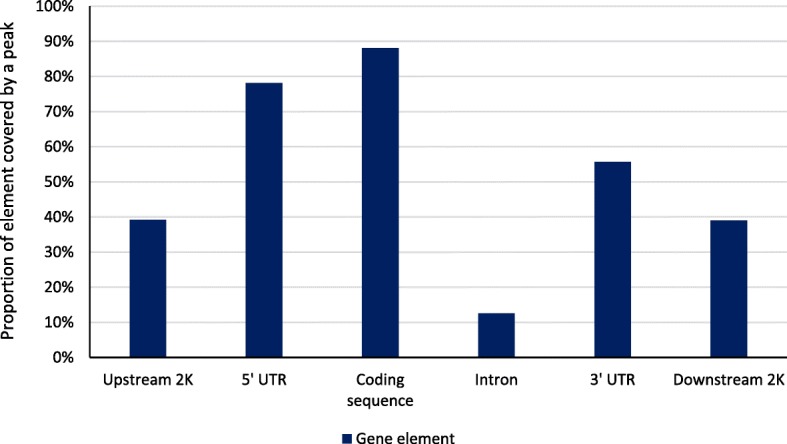
Proportion of genomic elements covered by a methylation peak. The percentage of total length for each gene element covered by a DNA methylation peak divided by the total length of each element in the genome for 2 kb upstream of the transcription start site, the 5′-untranslated region, the protein coding sequence, introns, the 3′-untranslated region, and 2 kb downstream of the transcription termination site

The proportion of the genome covered by methylation peaks ranged from 7.38% (cow F4I) to 10.52% (cow F1L) with an average of 8.43%. Average peak size ranged from 735 bp to 1080 bp for cows F3I and F1L, respectively. The proportion of gene element types covered by a peak is displayed in Fig. 4. The majority of coding sequence (86.8%) and 5′-untranslated region sequence (76.8%) resided within a methylation peak. In contrast, only 11.6% of intronic sequence was covered by a peak. Coordinates for peak regions are reported for each cow in Additional file 1: Table S1, Additional file 2: Table S2, Additional file 3: Table S3, Additional file 4: Table S4, Additional file 5: Table S5, and Additional file 6: Table S6.

### Geometric mean reads (GMR)

**Fig. 5:**
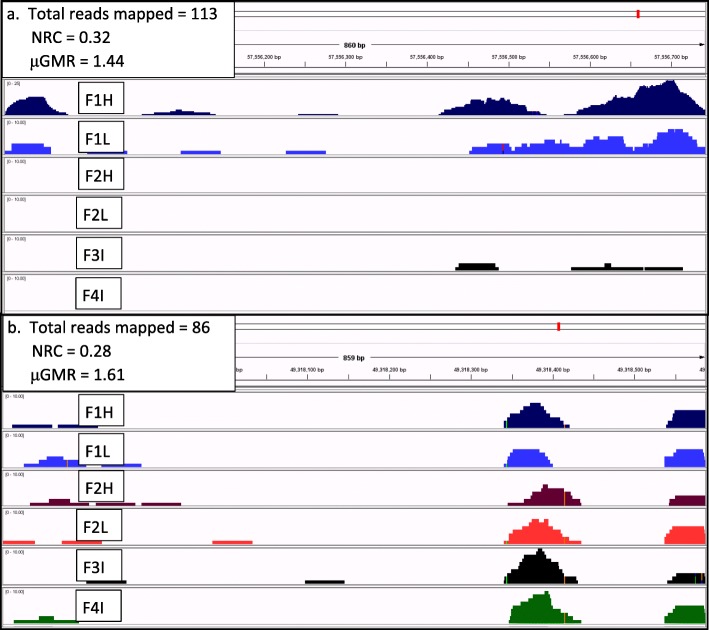
Average normalized reads count versus geometric means reads for two example regions. Individual cow alignments [23] for two ~ 860 bp regions. Panel **a**. demonstrates alignments for a region with high variability among cows on BTA19:57,555,882-57,556,746 and that is a significant environmental DMR, whereas panel **b**. demonstrates alignments for a region with minimal variation on BTA24:49,317,728-49,318,588. The identification of the cow corresponding to each panel is provided along with the total number of reads mapped to the region across all cows, the NRC, and μGMR

Following a visual inspection of results [24] and considering preliminary descriptive statistics, it was clear that reads were not normally distributed across cows or genomic regions. Skewed distributions were not unexpected as MeDIP enrichment preferentially targets GC rich regions because they are highly methylated and because of the existence of hypermethyated sites [25, 26]. Bias can also be introduced during library construction, particularly during PCR amplification of GC rich fragments [26]. Therefore, we derived a geometric mean reads (GMR) to describe general methylation patterns for this group of animals which is described in the methods. Figure 5 demonstrates alignment reads from two regions of the same length (~ 860 bp). Figure 5a demonstrates a region where two cows have a large number of reads, whereas Fig. 5b demonstrates a region with similar reads for all cows. The average normalized reads count (NRC) across these six cows was higher for the more variable region (0.32) than for the less variable region (0.28) despite four of the cows having more reads in the second region. The average GMR (μGMR) reflects that the majority of cows had more reads for the less variable region (μGMR = 1.61) than for the region with high variance (μGMR = 1.44).

**Fig. 6:**
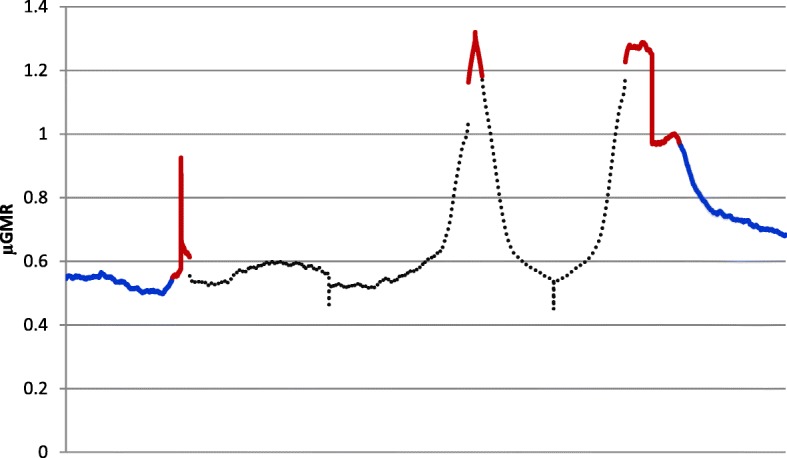
Average geometric means reads across 13,677 genes. Average geometric means reads (μGMR) 1 kb upstream and 1 kb downsteam (blue); first, middle, and last exons (red); and first and last introns (black) for 13,677 bovine genes

GMR of 13,677 unique *Bos taurus* protein coding genes downloaded from Ensembl [27] with completed coding sequence start and end coordinates were analyzed in the present study. μGMR for 1 kb upstream; first, middle and last exons; first and last intron; and 1 kb downstream are presented in Fig. 6 for these genes. Reads were generally low in the upstream region with the nadir μGMR occurring at 95 bp upstream of the TSS. μGMR was lower in the first exon than in later exons, particularly in the first half of initial exons. The middle exon tended to be most highly methylated, whereas the last exons were generally highly methylated at the beginning of the exon and had lower methylation in the second half of the exon. μGMR was highest for introns near the intron-exon junctions and were lowest in the middle and declined downstream as the distance from the gene increased.

### Genome wide methylation patterns

μGMR in non-overlapping 10 kb windows was determined to evaluate genome wide methylation patterns of uniquely mapped reads for all chromosomes (except for the Y chromosome) (see Additional file 7: Figure S1). Visually, there appeared to be fewer reads mapped to the centromeric ends of many chromosomes than mapped to the remainder of the chromosome. μGMR in the first 500 kb of the centromeric end (average of 0.81 ± 0.68 across all chromosomes) were less (*P <* 0.001) than the last 500 kb of the distal end (average of 1.06 ± 1.00) and middle of the chromosomes (average of 1.07 ± 0.51). These μGMR only represented uniquely mapped reads and not repetitive satellite sequences which would be expected to concentrate at the centromeric ends of the chromosome. μGMR for the distal end and middle of the chromosomes were not different, but μGMR was significantly less variable for middle of the chromosomes than for the centromeric and distal ends (*P <* 0.001). There were also large differences among chromosomes with the lowest for the X chromosome (μGMR = 0.64) and *Bos taurus* autosome (BTA) 6 (μGMR = 1.00), whereas BTA19 (μGMR = 1.25) and BTA27 (μGMR = 1.26) had the highest rates. The Spearman rank correlation between μGMR across 1 Mb windows with the number of protein coding genes in the window was 0.39 (*P <* 0.0001), indicating that higher gene density was associated with higher μGMR.

### Partially methylated domains (PMD)

Genomic regions with suppressed levels of methylation have been described as PMD [28], and such regions were apparent when evaluating μGMR across 10-kb windows. We used a permutation test to empirically identify PMD. There were 4725 PMD covering 511.2 Mb (~ 19%) of the genome identified, with 16% of autosomes and 66% of the X chromosome falling within a PMD. The largest single PMD was from 74.4 to 79.7 Mb on the X chromosome, whereas the largest autosomal PMD stretched from 72.5 to 76.7 Mb on BTA12.

There were 1153 genes located in significant PMD, including 898 autosomal genes and 255 on the X chromosome. This represented 6.8% of the 13,156 autosomal genes and 48.9% of the X genes. The PMD genes were submitted to DAVID [29, 30] for functional evaluation with 1021 matching known genes. There were 29 significant (FDR adjusted *P < 0.05*) functional annotation charts that are reported in Additional file 8: Table S7. Of those, 14 were part of an annotation cluster with an enrichment score of 31.91. The annotation charts for genes in that cluster are reported in Table 2 and encompassed 224 total genes, including 101 genes belonging to the *Olfactory Transduction* KEGG pathway [31].

**Table 2:** Significant functional annotation charts [29, 30] from the most highly enriched cluster for genes located within partially methylated domains

Category	Term	No. of genes	Fold Change	FDR
GOTERM_MF_DIRECT	*olfactory receptor activity*	112	4.4	5.70E-41
INTERPRO	*Olfactory receptor*	112	4.4	5.00E-40
UP_KEYWORDS	*Olfaction*	112	4.4	6.20E-40
UP_KEYWORDS	*Sensory transduction*	117	3.9	4.40E-37
GOTERM_MF_DIRECT	*G-protein coupled receptor activity*	119	3.5	2.50E-33
KEGG_PATHWAY	*Olfactory transduction*	101	3.8	5.20E-32
UP_KEYWORDS	*G-protein coupled receptor*	133	3.2	8.50E-32
INTERPRO	*GPCR, rhodopsin-like, 7TM*	134	3.2	9.60E-32
INTERPRO	*G protein-coupled receptor, rhodopsin-like*	133	3.2	9.80E-32
UP_KEYWORDS	*Transducer*	134	3.0	3.10E-29
GOTERM_BP_DIRECT	*G-protein coupled receptor signaling pathway*	93	3.4	9.30E-24
UP_KEYWORDS	*Receptor*	151	2.3	4.60E-21
UP_KEYWORDS	*Cell membrane*	143	1.9	5.30E-12
GOTERM_CC_DIRECT	*plasma membrane*	172	1.6	5.00E-08

### Putative differentially methylated regions (DMR)

There were 72 DMR with a significant false discovery rate (FDR; *P <* 0.05) identified by determining the fold change in a standardized number of reads in case versus control cows as described in the methods. The chromosome, starting position and ending position of the DMR are provided in Additional file 9: Table S8. An additional 252 environment specific DMR (Additional file 10: Table S9) were identified with 229 associated with farm 1, 24 associated with farm 2, and 1 that was associated with both herds. There were 2 regions that were identified as both case-control DMR and environmental DMR.

Additional file 11: Figure S2 (case-control) and Additional file 12: Figure S3 (environmental) show the most significant DMR within their broader genomic region of up to 40 kb from genomic elements that included known protein coding genes, pseudogenes, uncharacterized ncRNAs, and spliceosomal RNAs. In some instances, multiple putative DMR are located in close proximity. Additionally, the reads mapped to the third most significant environmental DMR represented the region with high variability in Fig. 5a. Genes (108) that were within 5 kb of a DMR were submitted to DAVID [29, 30] for functional evaluation with 96 matching known genes. Eight genes (listed Additional file 13: Table S10) were identified as part of two significant annotation charts of HOX (SMART) and homeobox (INTERPRO) with FDR adjusted *P <* 0.10.

## Discussion

Epigenetic descriptions in high yielding dairy cattle that have undergone intensive genetic selection can serve as a model to examine effects of selection on DNA methylation. We focused on DNA methylation in PBMC for this study because blood is accessible and farmers are unlikely to approve of invasive tissue sampling from the elite, high yielding cows that are of particular interest because they are population outliers. While blood is accessible, PBMC are also a mixed cell population which may make the epigenetic signal less clear; nevertheless, it was previously reported that CpG site variability in leukocytes could serve as an accurate predictor if CpG site variability in other tissues [32]. The large deviations of expected to actual milk, fat, and protein yield (Table 1) suggest that the differences in milk yield were not attributable to DNA sequence variation.

Many features of the cattle methylome reported here are shared with those from other species. The well described decline in promoter methylation [33, 34] (Fig. 2) was apparent. There is growing evidence that methylation is associated with alternative splicing across tissues [35] and that DNA methylation marks exon boundaries [36]. High levels of methylation across exons with a rapid decline of intronic methylation as distance from the exon-intron junction increased should be expected if methylation was a key exon marker. This was evident here with average methylation levels in the middle of introns as low as levels observed in the upstream region. Genes associated with the sensory perception of smell were highly enriched in human PMD [28]. We also found olfactory related genes to be enriched in PMD (Table 2), suggesting that PMD may be consistent across species. We noted a positive correlation between DNA methylation levels and gene density, which was previously reported for pigs [33] and in humans [37].

DNA methylation patterns from fetal and adult longissumus dorsi muscle were previously reported for Chinese Qinchuan cattle using a MeDIP-seq approach [34]. Average peak lengths were 974 (fetal) to 994 bp (adult) which are similar to our observations; a smaller proportion of the genome was reported to covered by a peak (4.4% for fetal and 4.6% for adult muscle) than was observed in the current study. They also observed more peaks aligned to regions with 5 to 10 bp per kb, whereas we observed higher levels between 15 and 20 bp. Reads mapped to gene elements were most likely to be aligned to an intron in both fetal and adult muscle, as we observed in the current study (Fig. 1). However, this was the result of the long length of introns in comparison to other gene elements. In contrast, a very small percentage of reads aligned to the 5′-untranslated region, but approximately 90% of 5′-untranslated region and coding sequences had a methylation peak both fetal and adult muscle tissue which is similar to what we observed in PBMC.

Higher rates of DNA methylation are reported for most genes on the inactive X chromosome when compared with the active X-chromosome [38], resulting in higher X-chromosome DNA methylation rates for females than for males [33]. However, hypermethylation of the inactive X is concentrated on promotors of silenced genes and other features of the X-chromosome may result in lower chromosome wide DNA methylation levels. The frequency of CpG islands on the human X was reported to be half of the genome-wide frequency, gene density is low on the X, and most X genes are relatively short [39].

We are unable to determine whether any of the DMR are directly responsible for phenotypic variation because a functional evaluation of epigenetic alterations is beyond the scope of this study and the transcriptomic data are not available for these individuals. For this reason, we refer to these regions as putative DMR. Nevertheless, there were regions that appear to harbor differential methylation and that have been previously associated with phenotypic variation in cattle. The putative DMR in the *SECTM1* region (Additional file 11: Figure S2 k) is intriguing because of *SECTM1* function and previously reported associations with cattle performance. Genes with roles in immune function are strong candidates for differential methylation in this study because we isolated DNA from PBMC. *SECTM1* is highly expressed in leukocytes [40] and *SECTM1* and *CD7* are reported to be *INF-γ* induced co-stimulators of T-cell proliferation [41]. *SECTM1* appears to have a role in in cattle immunity as there was a reported 2.73 fold increase in *SECTM1* expression in Angus cattle that were resistant to parasitic infection than in those that were susceptible [42]. Humans are reported to have a single *SECTM1* gene whereas there are multiple paralogs in cattle that have undergone positive selection [43]. The *60S ribosomal protein L7* pseudogene (Additional file 7: Figure S1f) was reported to have reduced expression in cattle with tuberculosis [44]. Additional regions have plausible relationships to differential methylation or immune function. For instance, the *NLK* pathway (Additional file 11: Figure S2c) is associated with transcriptional silencing via the methylation of *PPARG* target promoters at histone *H3K9* (http://www.uniprot.org/uniprot/Q9UBE8) [45], whereas three putative environmental DMR (Additional file 12: Figure S3a) are associated with *FCGR2B* which is associated with B-cell antibody production and immune complex phagocytosis (http://www.uniprot.org/uniprot/P31994) [45].

The functional annotation analysis indicated that homeobox genes were overrepresented in regions with DMR. Homeobox genes are reported to be differentially expressed in leukocytes [46]. However, caution is warranted when evaluating this pathway because of the relatively small number of genes [8] that were part of these functional charts.

## Conclusions

Evaluating DNA methylation in populations that have undergone intense genetic selection may further our understanding of the role of DNA methylation in population change. We describe DNA methylation patterns for dairy cows with extreme phenotypes for milk yield and report the existence of putative DMR with plausible, but unverified, relationships to phenotypic and performance. This reference methylome for high producing Holstein cows provides a resource to more fully evaluate such relationships between variation in DNA methylation and phenotypic variation.

## Methods

### Animals and blood samples

Ten mL of whole blood was obtained from the coccygeal vein of 6 lactating Holstein dairy cows located on 4 commercial Pennsylvania dairy farms (Pennsylvania State Institutional Animal Care and Use Committee protocol number 28889). Following centrifugation, the buffy coat was extracted and stored (− 20 °C) until DNA was extracted with a DNeasy® Blood & Tissue Kit (QIAGEN Sciences, Germantown, MD) per manufacturer instructions.

In order to identify commercial cows with extreme high milk yield phenotypes, we contacted 15 farms that participated in a milk testing program and that had herd average production approximately 50% greater than the average of Pennsylvania herds. The highest milk yielding cow from each herd and the poorest milk producing herd-mate of a similar age and parity were then selected and genotyped to generate genomic estimated breeding values (Table 1). Following genotyping, we selected two case-control pairs to maximize the likelihood that yield differences were due to epigenetic changes independent of DNA sequence variation. Additional potential case-control pairs were genotyped, but not selected for MeDIP-seq because yield deviations appeared to be explained largely by differences in genotype. The two intermediate production cows were also selected from the pool of high producing herds.

### MeDIP-seq and GMR

MeDIP-seq and a bioinformatics analysis was conducted by BGI (Shenzhen, China). Library construction followed a previously described protocol [47] and consisted of genomic DNA fragmentation (100–500 bp by sonication), 3′-A overhang and ligation of sequencing adaptors (Illumina Pair-End DNA Sample Prep Kit), denaturing of double-stranded DNA, immunoprecipitation via 5-mC antibody, and PCR amplification and size selection (200–300 bp, including adaptor sequence). Both methylated DNA controls and unmethylated DNA controls were used with each DNA sample to validate the pulldown procedure. Approximately 100 million paired-end reads were generated for each cow and the 49 bp ends were sequenced using an Illumina HiSeq 2000 sequencer. Sample libraries were indexed to facilitate multiplexed sequencing per lane [48].

Raw reads were processed to remove those containing 5′ or 3′ adapter sequences, unknown or low quality bases. The remaining clean reads were aligned to btau4.0 (http://hgdownload.cse.ucsc.edu/goldenPath/bosTau4/chromosomes) using the Short Oligonucleotide Analysis Package (v1 [49]). Whole genome peak scanning was conducted with MACS 1.4.0 (http://liulab.dfci.harvard.edu/MACS/) and only included reads with 2 or fewer mismatched bp. Peaks were aligned to btau4.0 and coordinates were then converted (https://genome.ucsc.edu/cgi-bin/hgLiftOver) to UMD 3.1 [50]. The analysis of repeats was conducted using RepeatMasker (http://www.repeatmasker.org) and repeat annotations were retrieved from the USCS database (http://hgdownload.cse.ucsc.edu/goldenPath/bosTau4/bigZips/bosTau4.fa.out.gz). CpG islands were regions of ≥200 bp, G + C content ≥50%, and the ratio of observed to expected CpG > 0.6 [51].

A second alignment to the current bovine assembly [50] was completed by Appistry, Inc. (St. Louis, MO, USA). The quality of reads in BAM files after alignment was evaluated with Picard (http://broadinstitute.github.io/picard/) and median quality scores ranged from 29 to 31. The number of reads per nucleotide (NR) was extracted using the mpileup option of SAMtools [52] with a minimum mapping quality ≥15. The GMR was determined as GMR = e^mean(ln(NR + 1)) – ln (2)/6^ in SAS (v 9.4; SAS Institute Inc., Cary, NC). The + 1 term was added so that the natural log could be derived for cows with no reads at a given nucleotide and the -ln (2)/6 partly removes the + 1 term and sets GMR = 1 if a single cow has a single read at a nucleotide. GMR was set to 0 if all 6 cows had no reads at a given nucleotide. A normalized reads count was calculated for the regions depicted in Fig. 1 as (RC*1,000,000)/(URC) where RC = the number of reads mapped to the region and URC = the total number of genome wide reads mapped to a unique sequence for each cow.

We standardized exon and intron lengths so that general methylation patterns for exons, introns, and exon-intron junctions could be described as opposed to non-specific methylation levels across the gene body. Exon lengths were standardized to the median exon length, which were 159 bp for first exons, 128 bp for all middle exons, and 503 bp for last exons. If there were more than 159 bp for the first exon, the first 79 bp were classified as nucleotides 1 to 79, the last 79 bp were classified as 81 to 159, and all others were classified as nucleotide 80. If there were fewer than 159 bp, the first 50% of nucleotides were associated with the first nucleotides of the exon, whereas the last 50% were associated with the last nucleotides of the exon. The same approach was used for the other exons and introns, with median intron lengths of 2616 bp for first introns and 1343 bp for last introns.

### Statistical analysis

Statistical analyses were performed with SAS. Differences in μGMR between the centromeric ends, middle, and distal ends of chromosomes were evaluated with the TTEST procedure. Likewise, tests of heterogeneous variance between the centromeric ends, middle, and distal ends of chromosomes were evaluated with the TTEST procedure.

### Identification of putative DMR

There were 1,223,424 regions with a length of 200 bp to 4199 bp (1,186,289 autosomal and 37,135 X chromosome) with regions defined as a continuous stretch of nucleotides with reads present for one or more cows; regions that were less than 200 bp apart were merged. Less than 1% of initial regions were > 4000 bp in length and those large regions were split into regions of 4000 bp with a restriction that the last segment had to be at least 200 bp in length; this resulted in 1514 regions with a length between 4000 bp and 4199 bp. The log_2_ of the total number of reads per region was determined for each cow and then standardized within cow to a mean of 100 and standard deviation of 5. We derived a relative standardized fold change to identify DMR for case versus control cows because t-tests identified regions with low variation among cows as significant even if the differences between case and controls were minimal. We used a standardized change because cows may have zero reads in a region preventing direct calculation of the ratio between case and control cows.

A permutation test was conducted to determine expectations for case-control and environmental distributions. Reads from a randomly selected region were drawn and then the two cows from farm 1 (F1H and F1L) were jointly assigned to either herd environment 1, herd environment 2, or herd environment 3 at random. Cows from farm 2 were then randomly assigned to one of the two remaining herd environments, with cows F3I and F4I assigned to the final environment. This resulted in 6 possible herd environment combinations. Within each herd environment, one cow was randomly assigned to be the high yield case cow, with the second cow from the environment serving as the control. This yielded 8 possible case-control combinations within each herd environment, for a total of 48 randomly assigned scenarios which are shown in Additional file 14: Table S11. This process was repeated 58,724,352 times, which was a rate of 48 samples per region with replacement. The actual number of times a region was randomly drawn ranged from 19 to 85.

The ratio of standardized reads for the randomly assigned case cow to the randomly assigned control was determined for herd environment 1 and herd environment 2, and the mean of those two ratios determined for each permutation. The ratio was not determined for the third herd environment because only data from two of the farms (farm 1 and farm 2) were case-control pairs. The mean (1.000) and standard deviation (0.0198) were then applied to the mean ratio of the two actual case-control pairs to determine the *P-Value* for the observed fold changes. *P-Values* were multiplied by 2 to account for the two-tailed aspect of the test. The *P-Value*s from all regions were then evaluated with the MULTTEST procedure of SAS to derive the genome-wide FDR.

To identify putative environment specific DMR, we first derived the mean of standardized reads for cows randomly assigned to herd environment 1, herd environment 2, and herd environment 3. The ratio of mean standardized reads for a single herd environment to the mean of standardized reads for all other cows was then derived for each permutation. The resulting mean (1.000) and standard deviation (0.0187) were then used to derive *P-Values* for ratios for farm 1 and farm 2. While the intermediate milk yield cows (F3I and F4I) were used to derive permutations, they were not from the same herd so were not considered when identifying putative environment specific DMR as at least two observations per environment are required [53]. A single dataset that contained *P-Values* from each herd comparison and from all regions (3,670,272 *P-Value*s) was then evaluated with the MULTTEST procedure of SAS to derive the genome-wide FDR.

Genes located within 5 kb of a DMR were submitted to DAVID [23] for functional evaluation with a medium classification stringency. There were 53 genes that fell within this range of a DMR and with a FDR adjusted *P <* 0.05. Therefore, we relaxed the FDR adjusted *P-Value* threshold to *P <* 0.10 which increased the number of genes to 108. The background genes were 13,677 unique bovine protein coding genes downloaded from Ensembl [23] which represented the pool of genes that we could detect overlapping DMR; of those, 12,980 were successfully converted to DAVID id numbers. We assumed default values which included medium classification stringency, and with annotation to three functional categories (COG_ONTOLOGY, UP_KEYWORDS, UP_SEQ_FEATURE), three gene ontology terms (GOTERM_BP_DIRECT, GOTERM_CC_DIRECT, GOTERM_MF_DIRECT), one pathway (KEGG_PATHWAY), and three protein domains (INTERPRO, PIR_SUPERFAMILY, SMART).

### Identification and evaluation of PMD

A permutation test was conducted to identify significant PMD. The genome was partitioned into non-overlapping 10 kb windows and the proportion of each window covered by the methylated regions described above was determined. A moving average of percent coverage for ten consecutive 10 kb windows was then determined. We drew ten windows at random (with replacement) with this process was repeated 1 million times. Based on the permutation test, the average percent coverage across ten consecutive 10 kb windows was expected to fall below 25% for 1% of ≥100 kb moving average windows if methylation levels in adjoining 10 kb windows are independent. Genomic regions with less than this amount were considered significant PMD at *P <* 0.01. Genes located within a PMD were submitted to DAVID [23] for functional evaluation using the same background genes and defaults as described for DMR.

## Additional files

Additional file 1: **Table S1.** Chromosome, start nucleotide, end nucleotide, length, summit, tags, Log *P*, and fold enrichment of significant peaks for Farm 1, case. DNA methylation peak information for Farm 1, case. (XLSX 14158 kb)

Additional file 2: **Table S2.** Chromosome, start nucleotide, end nucleotide, length, summit, tags, Log *P*, and fold enrichment of significant peaks for Farm 1, control. DNA methylation peak information for Farm 1, control. (XLSX 13541 kb)

Additional file 3: **Table S3.** Chromosome, start nucleotide, end nucleotide, length, summit, tags, Log *P*, and fold enrichment of significant peaks for Farm 2, case. DNA methylation peak information for Farm 2, case. (XLSX 14201 kb)

Additional file 4: **Table S4.** Chromosome, start nucleotide, end nucleotide, length, summit, tags, Log *P*, and fold enrichment of significant peaks for Farm 2, control. DNA methylation peak information for Farm 2, control. (XLSX 13499 kb)

Additional file 5: **Table S5.** Chromosome, start nucleotide, end nucleotide, length, summit, tags, Log *P*, and fold enrichment of significant peaks for Farm 3. DNA methylation peak information for Farm 3. (XLSX 14317 kb)

Additional file 6: **Table S6.** Chromosome, start nucleotide, end nucleotide, length, summit, tags, Log *P*, and fold enrichment of significant peaks for Farm 4. DNA methylation peak information for Farm 4. (XLSX 13513 kb)

Additional file 7: **Figure S1.** Average geometric mean reads per nucleotide over 10 kb windows and the number of annotated genes per Mb. Columns representing the μGMR of non-overlapping 10 kb windows for each chromosome and overlaid with an indication of the number of genes in each region. (PDF 1474 kb)

Additional file 8: **Table S7.** Functional annotation charts of genes located within partially methylated domains with the number of genes, fold enrichment, and FDR adjusted *P-Value* of the annotation chart. The functional category, term, number of genes, fold enrichment, and FDR adjusted *P-Value* of functional annotation charts with FDR adjusted *P <* 0.05. (DOCX 13 kb)

Additional file 9: **Table S8.** Location (chromosome, starting and ending nucleotide) of putative case-control differentially methylated regions, false discovery rate (FDR) adjusted *P-value*, and number of reads mapped to the region for case and control cows. The chromosome, starting nucleotide, ending nucleotide, FDR adjusted *P-Value*, and number of reads for each case and control cow for 72 regions with differential methylation. (DOCX 17 kb)

Additional file 10: **Table S9.** Location (chromosome, starting and ending nucleotide) of putative environmental differentially methylated regions, false discovery rate (FDR) adjusted *P-value*, and number of reads mapped to the region for all cows. The chromosome, starting nucleotide, ending nucleotide, FDR adjusted *P-Value*, and number of reads for each cow for 289 regions with differential methylation. (DOCX 29 kb)

Additional file 11: **Figure S2.** The location and surrounding genomic region for the top 11 most significant case-control differentially methylated regions. NCBI Genome Data Viewer of regions that harbor putative DMR with the location of each DMR and identification of nearby genes from NCBI *Bos taurus* Annotation Release 105, 2016-01-26. (PDF 294 kb)

Additional file 12: **Figure S3.** The location and surrounding genomic region for the top 10 most significant environmental differentially methylated regions. NCBI Genome Data Viewer of regions that harbor putative DMR with the location of each DMR and identification of nearby genes from NCBI *Bos taurus* Annotation Release 105, 2016-01-26. (PDF 119 kb)

Additional file 13: **Table S10.** Eight homeobox genes associated with a differentially methylated region (DMR) that were part of a single functional annotation chart with an FDR adjusted *P <* 0.05. Ensembl gene ID, gene name, location of DMR, and location of gene TSS. (DOCX 12 kb)

Additional file 14: **Table S11.** 48 sampling scenarios implemented in permutations to identify significance thresholds for identifying differentially methylated regions. A list of the high and low cow selected for each herd for each of the 48 sampling strategies. (DOCX 13 kb)

## Abbreviations

BTA: *Bos taurus* autosome
DMR: differentially methylated region
F1H: farm 1 high
F1L: farm 1 low
F2H: farm 2 high
F2L: farm 2 low
F3I: farm 3 intermediate
F4I: farm 4 intermediate
GMR: geometric mean reads
NR: number of reads per nucleotide
NRC: normalized reads count
PBMC: peripheral blood mononuclear cells
PMD: partially methylated domains
QTL: quantitative trait locus
RC: reads count
TSS: transcription start site
URC: unique reads count
μGMR: average geometric mean reads
