# Supplementary material for: DNA methylation patterns in peripheral blood mononuclear cells from Holstein cattle with variable milk yield
Source: BMC Genomics. 2018 Oct 11;19:744. doi: 10.1186/s12864-018-5124-9 (PMC6182825; doi:10.1186/s12864-018-5124-9)
Supplement: Supplementary file 7 — Figure S1. Average geometric mean reads per nucleotide over 10 kb windows and the number of annotated genes per Mb. Columns representing the μGMR of non-overlapping 10 kb windows for each chromosome and overlaid with an indication of the number of genes in each region. (PDF 1474 kb) [file 12864_2018_5124_MOESM7_ESM.pdf]

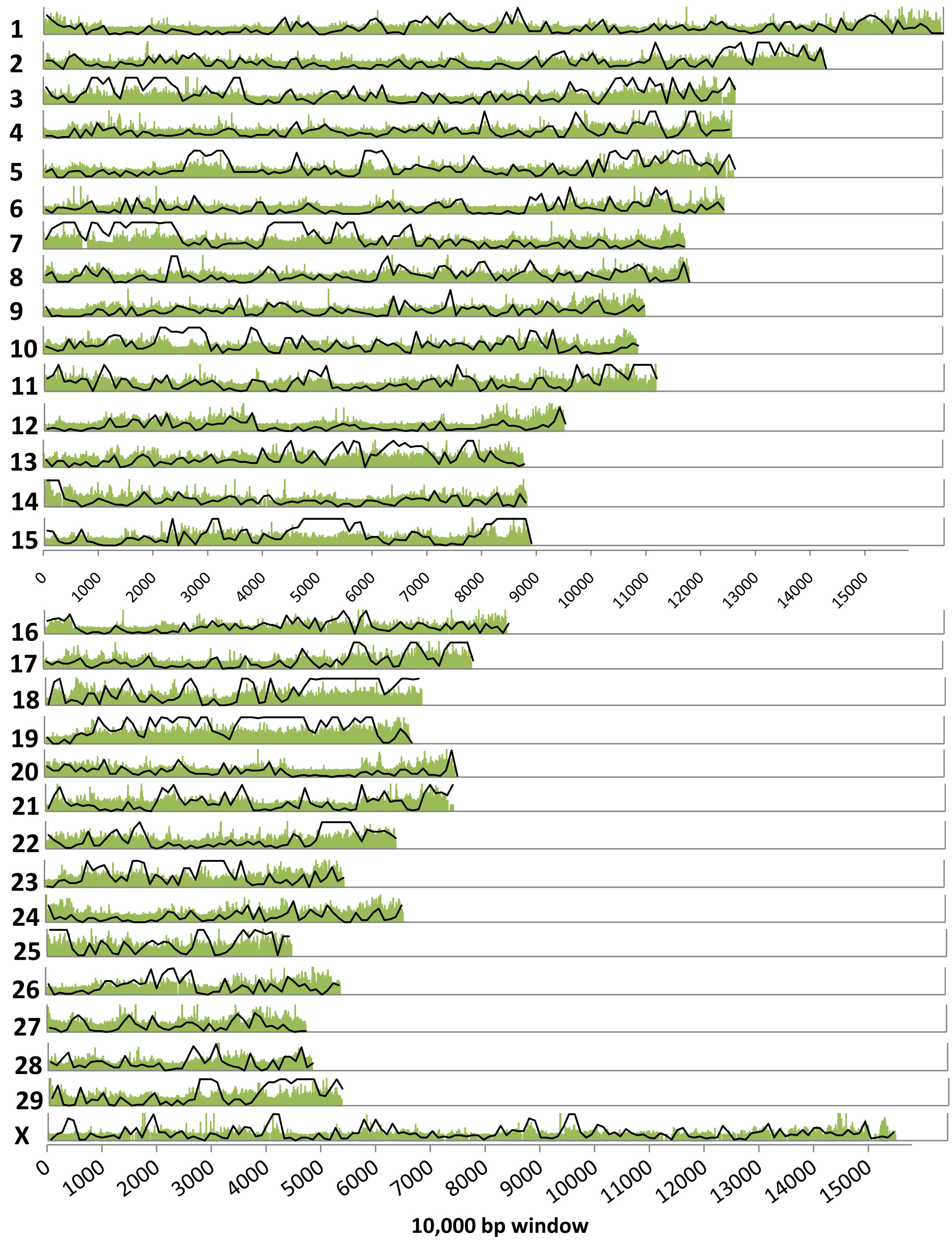

Figure S1. Average geometric mean reads per nucleotide (green bars; max=4) over 10 kb windows and the number of annotated genes per Mb (black line; max=26).
