# Supplementary material for: DNA methylation patterns in peripheral blood mononuclear cells from Holstein cattle with variable milk yield
Source: BMC Genomics. 2018 Oct 11;19:744. doi: 10.1186/s12864-018-5124-9 (PMC6182825; doi:10.1186/s12864-018-5124-9)
Supplement: Supplementary file 8 — Table S7. Functional annotation charts of genes located within partially methylated domains with the number of genes, fold enrichment, and FDR adjusted P-Value of the annotation chart. The functional category, term, number of genes, fold enrichment, and FDR adjusted P-Value of functional annotation charts with FDR adjusted P < 0.05. (DOCX 13 kb) [file 12864_2018_5124_MOESM8_ESM.docx]

Table S7. Functional annotation charts of genes located within partially methylated domains with the number of genes, fold enrichment, and FDR adjusted *P-Value* of the annotation chart.

| Category | Term | Number of genes | Fold Enrichment | FDR  *P-Value* |
| --- | --- | --- | --- | --- |
| GOTERM_MF_DIRECT | olfactory receptor activity | 112 | 4.4 | 5.70E-41 |
| INTERPRO | Olfactory receptor | 112 | 4.4 | 5.00E-40 |
| UP_KEYWORDS | Olfaction | 112 | 4.4 | 6.20E-40 |
| UP_KEYWORDS | Sensory transduction | 117 | 3.9 | 4.40E-37 |
| GOTERM_MF_DIRECT | G-protein coupled receptor activity | 119 | 3.5 | 2.50E-33 |
| KEGG_PATHWAY | Olfactory transduction | 101 | 3.8 | 5.20E-32 |
| UP_KEYWORDS | G-protein coupled receptor | 133 | 3.2 | 8.50E-32 |
| INTERPRO | GPCR, rhodopsin-like, 7TM | 134 | 3.2 | 9.60E-32 |
| INTERPRO | G protein-coupled receptor, rhodopsin-like | 133 | 3.2 | 9.80E-32 |
| UP_KEYWORDS | Transducer | 134 | 3 | 3.10E-29 |
| GOTERM_BP_DIRECT | G-protein coupled receptor signaling pathway | 93 | 3.4 | 9.30E-24 |
| UP_KEYWORDS | Receptor | 151 | 2.3 | 4.60E-21 |
| GOTERM_MF_DIRECT | odorant binding | 34 | 6.6 | 1.00E-16 |
| GOTERM_CC_DIRECT | integral component of membrane | 306 | 1.5 | 3.10E-13 |
| UP_KEYWORDS | Cell membrane | 143 | 1.9 | 5.30E-12 |
| SMART | SM01373 | 17 | 10.6 | 8.80E-11 |
| GOTERM_BP_DIRECT | sensory perception of smell | 32 | 4.7 | 3.30E-10 |
| INTERPRO | MAGE protein | 17 | 8.8 | 2.20E-09 |
| UP_KEYWORDS | Transmembrane | 345 | 1.4 | 3.70E-08 |
| GOTERM_CC_DIRECT | plasma membrane | 172 | 1.6 | 5.00E-08 |
| UP_KEYWORDS | Transmembrane helix | 343 | 1.4 | 6.60E-08 |
| INTERPRO | Melanoma associated antigen, MAGE, N-terminal | 10 | 8.7 | 4.80E-04 |
| INTERPRO | Transcription elongation factor A-like/Brain expressed X-linked-like | 8 | 11.6 | 7.90E-04 |
| UP_KEYWORDS | Membrane | 366 | 1.2 | 1.40E-03 |
| SMART | SM01392 | 8 | 9.6 | 6.50E-03 |
| SMART | LYZ1 | 8 | 8.9 | 1.20E-02 |
| PIR_SUPERFAMILY | brain-expressed X-linked protein | 6 | 12.5 | 1.70E-02 |
| INTERPRO | Brain-expressed X-linked protein | 6 | 13 | 2.40E-02 |
| INTERPRO | Glycoside hydrolase, family 22, lysozyme | 8 | 8 | 2.90E-02 |
