# Supplementary material for: DNA methylation patterns in peripheral blood mononuclear cells from Holstein cattle with variable milk yield
Source: BMC Genomics. 2018 Oct 11;19:744. doi: 10.1186/s12864-018-5124-9 (PMC6182825; doi:10.1186/s12864-018-5124-9)
Supplement: Supplementary file 9 — Table S8. Location (chromosome, starting and ending nucleotide) of putative case-control differentially methylated regions, false discovery rate (FDR) adjusted P-value, and number of reads mapped to the region for case and control cows. The chromosome, starting nucleotide, ending nucleotide, FDR adjusted P-Value, and number of reads for each case and control cow for 72 regions with differential methylation. (DOCX 17 kb) [file 12864_2018_5124_MOESM9_ESM.docx]

Table S8. Location^1^ (chromosome, starting and ending nucleotide) of putative case-control differentially methylated regions, false discovery rate (FDR) adjusted *P-value*, and number of reads mapped to the region for case and control cows.

| Chromosome | Starting Nucleotide | Ending Nucleotide | FDR P-Value | Farm 1  Case | Farm 2  Case | Farm 1  Control | Farm 2  Control |
| --- | --- | --- | --- | --- | --- | --- | --- |
| 10 | 90,937,269 | 90,937,898 | <0.00001 | 26 | 40 | 0 | 0 |
| X | 146,990,589 | 146,991,984 | 0.00001 | 226 | 254 | 0 | 131 |
| 19 | 20,228,019 | 20,228,264 | 0.00003 | 21 | 24 | 1 | 0 |
| 27 | 38,817,073 | 38,817,919 | 0.00035 | 0 | 0 | 31 | 25 |
| 23 | 37,498,776 | 37,499,554 | 0.00096 | 2 | 8 | 62 | 133 |
| 5 | 75,781,966 | 75,782,376 | 0.00102 | 11 | 21 | 0 | 0 |
| 4 | 72,610,271 | 72,610,981 | 0.00102 | 7 | 81 | 2 | 0 |
| 21 | 20,253,582 | 20,254,361 | 0.00110 | 17 | 17 | 0 | 0 |
| 2 | 64,395,760 | 64,396,041 | 0.00126 | 25 | 10 | 0 | 0 |
| 22 | 36,037,222 | 36,037,959 | 0.00126 | 3 | 0 | 64 | 28 |
| 19 | 51,059,864 | 51,060,239 | 0.00162 | 36 | 32 | 5 | 0 |
| 26 | 23,537,812 | 23,538,739 | 0.00229 | 18 | 42 | 4 | 0 |
| 10 | 27,733,614 | 27,734,496 | 0.00472 | 22 | 20 | 2 | 1 |
| 4 | 8,489,493 | 8,490,459 | 0.00577 | 8 | 22 | 0 | 0 |
| 27 | 5,895,733 | 5,896,043 | 0.00904 | 15 | 12 | 0 | 0 |
| 10 | 85,423,440 | 85,424,043 | 0.01081 | 9 | 16 | 0 | 0 |
| 6 | 38,449,783 | 38,450,933 | 0.01178 | 8 | 2 | 114 | 38 |
| 12 | 9,856,624 | 9,857,066 | 0.01185 | 62 | 61 | 34 | 0 |
| 8 | 97,127,116 | 97,127,681 | 0.01186 | 0 | 0 | 8 | 34 |
| 10 | 90,935,879 | 90,936,766 | 0.01518 | 18 | 8 | 0 | 0 |
| 14 | 79,188,908 | 79,190,350 | 0.01518 | 129 | 175 | 279 | 0 |
| 4 | 8,217,914 | 8,218,150 | 0.01533 | 0 | 0 | 16 | 16 |
| 4 | 9,163,540 | 9,163,847 | 0.01533 | 0 | 0 | 27 | 9 |
| 14 | 72,632,135 | 72,633,442 | 0.01533 | 10 | 13 | 0 | 0 |
| 5 | 75,806,225 | 75,807,326 | 0.01533 | 15 | 10 | 1 | 0 |
| 18 | 56,565,322 | 56,565,913 | 0.01533 | 13 | 9 | 0 | 0 |
| 11 | 8,570,859 | 8,571,199 | 0.01539 | 18 | 18 | 1 | 0 |
| 18 | 61,452,073 | 61,453,066 | 0.01557 | 0 | 0 | 19 | 15 |
| 5 | 94,363,578 | 94,364,911 | 0.01957 | 46 | 75 | 15 | 2 |
| 1 | 63,783,627 | 63,784,539 | 0.02074 | 15 | 7 | 0 | 0 |
| 11 | 31,896,320 | 31,897,289 | 0.02215 | 0 | 2 | 24 | 21 |
| 20 | 10,167,413 | 10,168,407 | 0.02215 | 0 | 1 | 46 | 12 |
| 8 | 45,176,243 | 45,177,105 | 0.02215 | 20 | 6 | 1 | 0 |
| 1 | 47,743,126 | 47,744,177 | 0.02242 | 4 | 0 | 52 | 16 |
| 13 | 38,173,722 | 38,174,973 | 0.02242 | 22 | 36 | 8 | 0 |
| 2 | 64,516,303 | 64,516,802 | 0.02356 | 19 | 16 | 3 | 0 |
| 9 | 82,563,124 | 82,564,255 | 0.02618 | 12 | 10 | 0 | 0 |
| 11 | 47,084,621 | 47,086,490 | 0.02618 | 5 | 1 | 114 | 22 |
| 19 | 51,053,006 | 51,053,419 | 0.02618 | 27 | 15 | 4 | 0 |
| 26 | 21,746,108 | 21,747,548 | 0.02785 | 15 | 36 | 6 | 0 |
| 1 | 121,617,572 | 121,618,375 | 0.02855 | 30 | 36 | 1 | 4 |
| 14 | 56,651,567 | 56,652,258 | 0.02855 | 6 | 18 | 0 | 0 |
| 6 | 69,062,780 | 69,064,381 | 0.02855 | 9 | 14 | 0 | 0 |
| 8 | 39,366,672 | 39,367,758 | 0.02855 | 7 | 14 | 0 | 1 |
| 13 | 43,112,930 | 43,113,484 | 0.02855 | 8 | 14 | 0 | 0 |
| 9 | 43,939,124 | 43,939,620 | 0.02855 | 14 | 8 | 0 | 0 |
| 15 | 60,563,402 | 60,563,780 | 0.02855 | 14 | 8 | 0 | 0 |
| 21 | 36,496,362 | 36,498,282 | 0.02855 | 54 | 31 | 21 | 0 |
| 19 | 58,604,424 | 58,605,375 | 0.03376 | 16 | 16 | 2 | 0 |
| 8 | 61,550,439 | 61,551,531 | 0.03377 | 0 | 0 | 23 | 8 |
| 14 | 79,212,320 | 79,214,056 | 0.03443 | 99 | 108 | 161 | 0 |
| 24 | 25,718,297 | 25,719,400 | 0.03838 | 11 | 8 | 131 | 73 |
| 16 | 76,812,613 | 76,813,359 | 0.03912 | 2 | 2 | 34 | 29 |
| 2 | 28,378,139 | 28,378,806 | 0.04084 | 0 | 0 | 30 | 7 |
| 14 | 32,568,038 | 32,568,530 | 0.04212 | 24 | 10 | 1 | 0 |
| 29 | 22,738,759 | 22,740,584 | 0.04212 | 24 | 66 | 9 | 3 |
| 27 | 5,698,602 | 5,699,048 | 0.04212 | 39 | 11 | 0 | 4 |
| 14 | 79,200,193 | 79,201,937 | 0.04212 | 88 | 120 | 176 | 0 |
| 6 | 89,565,969 | 89,566,956 | 0.04212 | 10 | 10 | 0 | 0 |
| 9 | 24,932,817 | 24,933,697 | 0.04212 | 10 | 10 | 0 | 0 |
| 15 | 46,478,105 | 46,478,430 | 0.04212 | 10 | 10 | 0 | 0 |
| 23 | 43,328,349 | 43,328,947 | 0.04212 | 9 | 10 | 1 | 1 |
| 23 | 45,504,095 | 45,505,417 | 0.04212 | 10 | 10 | 0 | 0 |
| 16 | 41,305,125 | 41,306,187 | 0.04440 | 0 | 0 | 12 | 13 |
| 14 | 79,173,780 | 79,176,411 | 0.04440 | 62 | 105 | 106 | 0 |
| 1 | 129,908,927 | 129,909,462 | 0.04440 | 1 | 0 | 14 | 12 |
| 5 | 38,881,427 | 38,882,032 | 0.04440 | 0 | 0 | 13 | 12 |
| 24 | 11,858,927 | 11,859,485 | 0.04440 | 0 | 0 | 14 | 12 |
| 15 | 64,354,314 | 64,354,931 | 0.04566 | 8 | 13 | 0 | 0 |
| 15 | 44,840,651 | 44,841,354 | 0.04568 | 12 | 8 | 0 | 0 |
| 18 | 63,346,501 | 63,347,197 | 0.04747 | 99 | 65 | 0 | 83 |
| 2 | 85,024,252 | 85,024,915 | 0.04958 | 16 | 22 | 3 | 0 |

^1^Aligned to: UMD_3.1 (<http://www.ncbi.nlm.nih.gov/assembly/GCA_000003055.4>)
