# Supplementary material for: DNA methylation patterns in peripheral blood mononuclear cells from Holstein cattle with variable milk yield
Source: BMC Genomics. 2018 Oct 11;19:744. doi: 10.1186/s12864-018-5124-9 (PMC6182825; doi:10.1186/s12864-018-5124-9)
Supplement: Supplementary file 10 — Table S9. Location (chromosome, starting and ending nucleotide) of putative environmental differentially methylated regions, false discovery rate (FDR) adjusted P-value, and number of reads mapped to the region for all cows. The chromosome, starting nucleotide, ending nucleotide, FDR adjusted P-Value, and number of reads for each cow for 289 regions with differential methylation. (DOCX 29 kb) [file 12864_2018_5124_MOESM10_ESM.docx]

Table S9. Location^1^ (chromosome, starting and ending nucleotide) of putative environmental differentially methylated regions, false discovery rate (FDR) adjusted *P-value*, and number of reads mapped to the region for all cows.

| Chromosome | Starting Nucleotide | Ending Nucleotide | FDR P-Value | Farm 1  Case | Farm 1  Control | Farm 2  Case | Farm 2  Control | Farm 3 | Farm 4 |
| --- | --- | --- | --- | --- | --- | --- | --- | --- | --- |
| 3 | 7959043 | 7959802 | <0.00001 | 0 | 0 | 106 | 33 | 50 | 79 |
| 3 | 7965689 | 7966743 | <0.00001 | 1 | 0 | 115 | 51 | 66 | 116 |
| 19 | 57555882 | 57556746 | <0.00001 | 81 | 27 | 0 | 0 | 5 | 0 |
| 25 | 36133080 | 36133784 | 0.00001 | 12 | 0 | 44 | 63 | 68 | 136 |
| 20 | 69482161 | 69482790 | 0.00033 | 2 | 0 | 28 | 14 | 28 | 36 |
| 3 | 7960093 | 7961374 | 0.00033 | 0 | 0 | 29 | 4 | 13 | 42 |
| X | 114461670 | 114462525 | 0.00033 | 0 | 1 | 31 | 11 | 35 | 36 |
| 17 | 5462594 | 5463357 | 0.00047 | 21 | 35 | 0 | 0 | 19 | 29 |
| 15 | 50741615 | 50742486 | 0.00048 | 22 | 42 | 0 | 0 | 17 | 21 |
| 23 | 44219355 | 44219971 | 0.00075 | 1 | 0 | 26 | 15 | 21 | 33 |
| 5 | 114621947 | 114622583 | 0.00105 | 4 | 0 | 21 | 24 | 21 | 56 |
| 8 | 15046213 | 15047498 | 0.00113 | 48 | 54 | 2 | 0 | 5 | 4 |
| 23 | 51106551 | 51106837 | 0.00129 | 1 | 3 | 10 | 38 | 28 | 18 |
| 20 | 66888430 | 66888659 | 0.00155 | 2 | 0 | 31 | 23 | 19 | 16 |
| 28 | 35218320 | 35219157 | 0.00158 | 7 | 2 | 45 | 42 | 47 | 50 |
| 23 | 27682555 | 27684229 | 0.00158 | 68 | 70 | 1 | 5 | 130 | 51 |
| 21 | 67092138 | 67092829 | 0.00158 | 0 | 5 | 11 | 55 | 24 | 32 |
| 10 | 58314131 | 58314860 | 0.00158 | 15 | 0 | 62 | 50 | 77 | 17 |
| 19 | 63057003 | 63057595 | 0.00158 | 2 | 2 | 34 | 22 | 27 | 42 |
| 7 | 47350214 | 47352056 | 0.00158 | 7 | 0 | 18 | 26 | 38 | 49 |
| 10 | 77407046 | 77407879 | 0.00158 | 3 | 0 | 18 | 32 | 23 | 34 |
| X | 146990589 | 146991984 | 0.00197 | 226 | 0 | 254 | 131 | 241 | 178 |
| 1 | 97650244 | 97651261 | 0.00197 | 0 | 2 | 24 | 20 | 14 | 22 |
| 7 | 54631568 | 54633190 | 0.00233 | 11 | 0 | 27 | 36 | 43 | 47 |
| 18 | 53080750 | 53081182 | 0.00237 | 9 | 0 | 19 | 39 | 38 | 41 |
| 15 | 54344547 | 54345131 | 0.00264 | 37 | 58 | 2 | 0 | 3 | 3 |
| 15 | 48752675 | 48753562 | 0.00264 | 46 | 22 | 0 | 0 | 15 | 7 |
| 23 | 25423479 | 25423920 | 0.00264 | 0 | 0 | 20 | 13 | 9 | 11 |
| 17 | 71679194 | 71680067 | 0.00264 | 9 | 0 | 28 | 44 | 25 | 51 |
| 19 | 42426519 | 42426949 | 0.00264 | 0 | 31 | 19 | 38 | 0 | 0 |
| 5 | 116682041 | 116682578 | 0.00264 | 10 | 2 | 44 | 55 | 70 | 46 |
| 19 | 24991090 | 24991764 | 0.00301 | 0 | 2 | 20 | 14 | 16 | 26 |
| 24 | 6302379 | 6302704 | 0.00351 | 0 | 0 | 7 | 29 | 9 | 11 |
| 16 | 52538403 | 52538781 | 0.00351 | 0 | 0 | 10 | 11 | 8 | 20 |
| 7 | 21915354 | 21915680 | 0.00351 | 5 | 0 | 17 | 35 | 27 | 34 |
| 17 | 6108412 | 6108809 | 0.00351 | 2 | 0 | 19 | 25 | 20 | 9 |
| 18 | 55551219 | 55551609 | 0.00351 | 4 | 0 | 16 | 26 | 21 | 30 |
| 18 | 63273303 | 63273623 | 0.00351 | 23 | 0 | 59 | 44 | 75 | 34 |
| 23 | 27712384 | 27713296 | 0.00447 | 28 | 82 | 0 | 2 | 31 | 8 |
| 7 | 98468522 | 98469261 | 0.00457 | 0 | 47 | 61 | 45 | 115 | 84 |
| 19 | 50328029 | 50329876 | 0.00497 | 8 | 5 | 60 | 57 | 66 | 78 |
| X | 38039079 | 38039342 | 0.00497 | 6 | 7 | 36 | 48 | 70 | 86 |
| 2 | 136228377 | 136228599 | 0.00562 | 0 | 0 | 5 | 24 | 12 | 7 |
| 21 | 66029868 | 66030577 | 0.00667 | 1 | 0 | 9 | 16 | 7 | 14 |
| 17 | 72432595 | 72433968 | 0.00679 | 22 | 0 | 60 | 24 | 42 | 82 |
| 4 | 89962064 | 89962613 | 0.00694 | 3 | 0 | 67 | 22 | 2 | 23 |
| 27 | 26381511 | 26382384 | 0.00708 | 226 | 84 | 1001 | 1039 | 1045 | 2013 |
| 5 | 118766762 | 118767930 | 0.00732 | 2 | 23 | 76 | 65 | 62 | 68 |
| 5 | 31013075 | 31013675 | 0.00781 | 10 | 2 | 44 | 33 | 60 | 55 |
| 2 | 63109435 | 63110444 | 0.00864 | 2 | 2 | 15 | 31 | 24 | 27 |
| 13 | 79845515 | 79845893 | 0.00864 | 3 | 1 | 19 | 28 | 17 | 21 |
| 28 | 33817817 | 33818519 | 0.00864 | 10 | 0 | 42 | 25 | 30 | 30 |
| 17 | 68918221 | 68918566 | 0.00877 | 0 | 0 | 5 | 12 | 10 | 16 |
| 18 | 63334193 | 63336402 | 0.00894 | 40 | 0 | 54 | 56 | 5 | 8 |
| 14 | 83087730 | 83088660 | 0.00913 | 76 | 145 | 32 | 0 | 0 | 57 |
| 8 | 57548474 | 57548807 | 0.00913 | 8 | 0 | 27 | 17 | 36 | 38 |
| 3 | 107779366 | 107779934 | 0.00924 | 0 | 0 | 4 | 15 | 9 | 22 |
| 2 | 131950060 | 131950528 | 0.00926 | 14 | 5 | 65 | 76 | 80 | 90 |
| 11 | 75517822 | 75519422 | 0.00934 | 38 | 2 | 70 | 121 | 62 | 108 |
| 13 | 70493126 | 70494294 | 0.00946 | 3 | 0 | 16 | 10 | 19 | 50 |
| 8 | 63527416 | 63528329 | 0.0095 | 6 | 2 | 34 | 36 | 33 | 41 |
| 20 | 58887638 | 58888164 | 0.00964 | 6 | 5 | 57 | 48 | 42 | 34 |
| 10 | 21330348 | 21331599 | 0.01072 | 13 | 0 | 20 | 36 | 36 | 57 |
| 18 | 7815127 | 7815647 | 0.01072 | 2 | 4 | 20 | 58 | 22 | 28 |
| 14 | 4567513 | 4568795 | 0.01072 | 13 | 3 | 44 | 42 | 49 | 58 |
| 3 | 113751333 | 113751981 | 0.01076 | 0 | 1 | 8 | 10 | 24 | 28 |
| 19 | 39731221 | 39732157 | 0.01076 | 2 | 4 | 26 | 28 | 34 | 31 |
| 13 | 53682099 | 53682873 | 0.01076 | 6 | 0 | 19 | 17 | 24 | 35 |
| 18 | 48892755 | 48893698 | 0.01076 | 4 | 0 | 16 | 19 | 16 | 32 |
| 7 | 42771793 | 42772245 | 0.01179 | 0 | 0 | 12 | 20 | 6 | 6 |
| 22 | 57799392 | 57799843 | 0.01179 | 9 | 3 | 37 | 40 | 40 | 56 |
| 29 | 12333787 | 12334992 | 0.01196 | 0 | 3 | 18 | 18 | 10 | 16 |
| 7 | 46630929 | 46631546 | 0.01196 | 5 | 0 | 25 | 16 | 12 | 31 |
| 23 | 7823981 | 7824432 | 0.01196 | 11 | 0 | 19 | 21 | 44 | 44 |
| 7 | 21002171 | 21002576 | 0.01196 | 2 | 0 | 10 | 18 | 16 | 18 |
| 5 | 56048947 | 56050687 | 0.012 | 18 | 2 | 78 | 24 | 62 | 90 |
| 14 | 79510846 | 79511219 | 0.012 | 0 | 0 | 19 | 12 | 14 | 1 |
| 14 | 2211525 | 2212226 | 0.01201 | 2 | 0 | 14 | 12 | 14 | 21 |
| 5 | 106757357 | 106757713 | 0.01201 | 8 | 1 | 22 | 28 | 26 | 28 |
| 14 | 1526821 | 1528176 | 0.01306 | 2 | 1 | 17 | 28 | 28 | 20 |
| 3 | 12332365 | 12332718 | 0.01315 | 0 | 0 | 4 | 7 | 12 | 19 |
| 5 | 107035543 | 107036370 | 0.0148 | 7 | 3 | 39 | 80 | 32 | 47 |
| 9 | 73235274 | 73235585 | 0.01518 | 0 | 0 | 1 | 13 | 21 | 12 |
| 21 | 28024316 | 28024774 | 0.01518 | 0 | 5 | 13 | 26 | 11 | 26 |
| 13 | 58220249 | 58220796 | 0.01518 | 18 | 3 | 47 | 49 | 61 | 69 |
| 15 | 13434243 | 13435209 | 0.01518 | 0 | 0 | 6 | 10 | 16 | 7 |
| 11 | 103759414 | 103759971 | 0.01522 | 4 | 5 | 38 | 37 | 36 | 31 |
| 14 | 15264799 | 15265700 | 0.01522 | 10 | 3 | 36 | 59 | 42 | 78 |
| 13 | 27925627 | 27926138 | 0.01542 | 30 | 32 | 0 | 2 | 28 | 10 |
| 29 | 48031277 | 48032169 | 0.01567 | 4 | 11 | 44 | 48 | 69 | 66 |
| 16 | 53717795 | 53718418 | 0.01567 | 0 | 0 | 6 | 16 | 2 | 26 |
| 13 | 83774634 | 83774902 | 0.01567 | 12 | 1 | 34 | 52 | 51 | 42 |
| 25 | 1201996 | 1203286 | 0.01567 | 4 | 0 | 18 | 8 | 26 | 30 |
| 25 | 35007086 | 35007824 | 0.01573 | 12 | 0 | 20 | 32 | 29 | 42 |
| 16 | 52651040 | 52651922 | 0.01632 | 12 | 0 | 33 | 24 | 26 | 38 |
| 25 | 2528716 | 2529264 | 0.01644 | 0 | 0 | 6 | 10 | 12 | 9 |
| 18 | 11525934 | 11526255 | 0.01653 | 2 | 1 | 11 | 12 | 10 | 28 |
| 19 | 29538097 | 29538573 | 0.01653 | 10 | 27 | 0 | 0 | 10 | 17 |
| 19 | 53447776 | 53448134 | 0.01683 | 4 | 0 | 21 | 24 | 15 | 16 |
| 24 | 61322325 | 61322552 | 0.01692 | 0 | 4 | 18 | 24 | 11 | 20 |
| 14 | 65074516 | 65074791 | 0.01692 | 0 | 0 | 10 | 4 | 11 | 13 |
| 2 | 128660421 | 128661129 | 0.01692 | 3 | 0 | 12 | 22 | 20 | 19 |
| X | 144093650 | 144094050 | 0.01713 | 0 | 2 | 26 | 19 | 8 | 9 |
| 25 | 40862574 | 40862950 | 0.01713 | 1 | 0 | 5 | 12 | 5 | 29 |
| 3 | 10973092 | 10973507 | 0.01713 | 0 | 0 | 17 | 2 | 12 | 14 |
| X | 4807806 | 4808243 | 0.01713 | 3 | 5 | 24 | 34 | 33 | 38 |
| 12 | 87508020 | 87508556 | 0.01713 | 29 | 0 | 41 | 31 | 0 | 10 |
| 23 | 9193558 | 9194027 | 0.01767 | 8 | 0 | 17 | 29 | 25 | 27 |
| 7 | 41718450 | 41719492 | 0.01796 | 14 | 0 | 32 | 28 | 41 | 28 |
| 5 | 11977765 | 11978461 | 0.0181 | 27 | 29 | 1 | 0 | 1 | 2 |
| 13 | 78603039 | 78603272 | 0.01873 | 5 | 1 | 15 | 19 | 18 | 22 |
| 16 | 42485660 | 42486147 | 0.01931 | 1 | 0 | 12 | 16 | 12 | 15 |
| 13 | 48579476 | 48580004 | 0.02015 | 5 | 0 | 14 | 30 | 20 | 22 |
| 14 | 8960597 | 8961654 | 0.02015 | 4 | 0 | 9 | 20 | 27 | 18 |
| 18 | 57310315 | 57310639 | 0.02015 | 0 | 0 | 5 | 11 | 13 | 13 |
| 13 | 82387682 | 82388160 | 0.02042 | 9 | 1 | 18 | 25 | 20 | 36 |
| 2 | 130865994 | 130866830 | 0.02042 | 14 | 0 | 31 | 37 | 20 | 43 |
| 7 | 20466139 | 20467111 | 0.02071 | 3 | 3 | 33 | 3 | 32 | 62 |
| 16 | 53310150 | 53310807 | 0.02109 | 2 | 0 | 12 | 12 | 11 | 21 |
| 3 | 3692188 | 3694830 | 0.02109 | 9 | 0 | 31 | 10 | 28 | 37 |
| 29 | 34307702 | 34308698 | 0.02113 | 45 | 9 | 0 | 0 | 6 | 12 |
| 7 | 18336516 | 18337515 | 0.02113 | 5 | 2 | 28 | 13 | 27 | 48 |
| 7 | 16005335 | 16005926 | 0.02113 | 0 | 0 | 2 | 10 | 12 | 18 |
| 4 | 114999748 | 115000604 | 0.02116 | 2 | 2 | 9 | 28 | 17 | 40 |
| 15 | 82561365 | 82561776 | 0.02259 | 3 | 1 | 22 | 24 | 26 | 32 |
| 18 | 25028160 | 25029525 | 0.02259 | 18 | 0 | 13 | 48 | 41 | 51 |
| 16 | 45000327 | 45001071 | 0.02329 | 8 | 4 | 35 | 42 | 36 | 62 |
| 3 | 14323197 | 14323802 | 0.02329 | 6 | 1 | 30 | 35 | 20 | 39 |
| 18 | 11744782 | 11746195 | 0.02329 | 3 | 0 | 9 | 12 | 13 | 23 |
| 18 | 6606154 | 6606497 | 0.0236 | 19 | 7 | 68 | 76 | 63 | 90 |
| 22 | 49691207 | 49692046 | 0.0236 | 6 | 2 | 15 | 44 | 24 | 46 |
| 21 | 34851372 | 34851962 | 0.0236 | 6 | 7 | 25 | 44 | 48 | 74 |
| 25 | 15246856 | 15247725 | 0.0236 | 14 | 22 | 1 | 0 | 1 | 0 |
| 17 | 72791113 | 72791449 | 0.0236 | 2 | 0 | 15 | 14 | 16 | 11 |
| 7 | 20344025 | 20344789 | 0.0236 | 9 | 0 | 22 | 22 | 24 | 24 |
| 17 | 67361975 | 67362939 | 0.0236 | 15 | 11 | 62 | 96 | 82 | 86 |
| 3 | 67576076 | 67576650 | 0.0236 | 17 | 11 | 0 | 0 | 24 | 9 |
| 15 | 57268167 | 57269234 | 0.0236 | 3 | 6 | 22 | 44 | 48 | 43 |
| 9 | 104826906 | 104827328 | 0.02399 | 29 | 2 | 39 | 74 | 72 | 75 |
| 19 | 58137145 | 58138186 | 0.02439 | 13 | 1 | 34 | 30 | 27 | 31 |
| 19 | 45502177 | 45503415 | 0.02439 | 6 | 5 | 30 | 43 | 55 | 54 |
| 18 | 13810216 | 13810603 | 0.02439 | 0 | 1 | 18 | 10 | 12 | 15 |
| 14 | 3508212 | 3508806 | 0.02439 | 3 | 4 | 15 | 36 | 25 | 32 |
| 9 | 94757744 | 94758436 | 0.02439 | 4 | 1 | 19 | 23 | 24 | 37 |
| 17 | 73969790 | 73970398 | 0.02439 | 6 | 0 | 9 | 20 | 33 | 25 |
| 18 | 11290102 | 11290593 | 0.02439 | 0 | 0 | 5 | 6 | 9 | 13 |
| 23 | 49939178 | 49939904 | 0.02446 | 15 | 7 | 0 | 0 | 28 | 8 |
| 28 | 13343381 | 13344315 | 0.02446 | 19 | 5 | 57 | 67 | 60 | 65 |
| 13 | 28656248 | 28657213 | 0.02463 | 6 | 2 | 22 | 50 | 26 | 26 |
| 25 | 33415448 | 33415815 | 0.02463 | 5 | 3 | 18 | 43 | 32 | 40 |
| 23 | 15863229 | 15863597 | 0.02489 | 0 | 0 | 8 | 8 | 8 | 9 |
| 25 | 35003101 | 35003483 | 0.02494 | 7 | 0 | 15 | 24 | 25 | 26 |
| 2 | 133504556 | 133505015 | 0.02538 | 5 | 0 | 13 | 21 | 17 | 20 |
| 2 | 134332144 | 134333327 | 0.02543 | 8 | 0 | 18 | 31 | 20 | 22 |
| 17 | 56971981 | 56972340 | 0.02582 | 1 | 0 | 10 | 20 | 13 | 10 |
| 19 | 51478552 | 51479168 | 0.02604 | 3 | 2 | 10 | 28 | 36 | 36 |
| 13 | 17675291 | 17676125 | 0.02672 | 6 | 0 | 31 | 6 | 29 | 28 |
| 7 | 13500130 | 13500494 | 0.02672 | 0 | 0 | 10 | 8 | 5 | 10 |
| 23 | 28846475 | 28846887 | 0.02672 | 15 | 36 | 2 | 0 | 21 | 15 |
| 19 | 33776186 | 33777183 | 0.02753 | 2 | 3 | 20 | 29 | 16 | 40 |
| 23 | 6573145 | 6573434 | 0.02753 | 5 | 1 | 35 | 15 | 31 | 51 |
| 11 | 74376396 | 74376717 | 0.02947 | 0 | 0 | 5 | 13 | 4 | 13 |
| 9 | 95328379 | 95329481 | 0.02957 | 2 | 4 | 10 | 32 | 27 | 41 |
| X | 13692711 | 13693589 | 0.02957 | 5 | 0 | 18 | 12 | 16 | 39 |
| 10 | 87871369 | 87871696 | 0.02968 | 17 | 28 | 0 | 2 | 3 | 0 |
| 20 | 71335332 | 71336237 | 0.03042 | 1 | 0 | 8 | 12 | 13 | 22 |
| 13 | 76987897 | 76988599 | 0.03042 | 5 | 3 | 45 | 38 | 36 | 29 |
| 11 | 83199223 | 83199621 | 0.031 | 1 | 0 | 16 | 19 | 6 | 14 |
| 7 | 67861638 | 67862087 | 0.03111 | 32 | 9 | 0 | 0 | 7 | 12 |
| 16 | 79353112 | 79354318 | 0.03147 | 14 | 3 | 42 | 52 | 41 | 37 |
| 21 | 19672662 | 19673007 | 0.03202 | 1 | 0 | 6 | 20 | 15 | 15 |
| 21 | 65789978 | 65790685 | 0.03202 | 32 | 4 | 82 | 109 | 75 | 60 |
| 25 | 41886896 | 41887325 | 0.03202 | 3 | 0 | 9 | 16 | 16 | 12 |
| 25 | 1972891 | 1973822 | 0.03202 | 1 | 2 | 19 | 12 | 16 | 33 |
| 21 | 8117381 | 8118237 | 0.03202 | 7 | 0 | 10 | 31 | 28 | 14 |
| 11 | 71287689 | 71288095 | 0.03202 | 6 | 1 | 13 | 20 | 15 | 38 |
| 25 | 601161 | 602570 | 0.03205 | 0 | 2 | 6 | 16 | 12 | 20 |
| 20 | 69988420 | 69989116 | 0.03217 | 6 | 3 | 22 | 37 | 21 | 40 |
| 16 | 1605500 | 1605931 | 0.03263 | 5 | 27 | 0 | 0 | 0 | 0 |
| X | 34433761 | 34434282 | 0.03301 | 7 | 2 | 17 | 48 | 21 | 36 |
| 22 | 52378127 | 52378363 | 0.03301 | 14 | 21 | 0 | 1 | 1 | 3 |
| 24 | 4260725 | 4261511 | 0.03309 | 15 | 0 | 26 | 30 | 35 | 30 |
| 13 | 70055873 | 70057024 | 0.03309 | 17 | 30 | 0 | 0 | 1 | 15 |
| 11 | 103273057 | 103273652 | 0.03335 | 0 | 2 | 12 | 12 | 14 | 12 |
| 25 | 9370130 | 9370613 | 0.03352 | 4 | 2 | 26 | 37 | 0 | 24 |
| 5 | 110232910 | 110233175 | 0.03352 | 2 | 0 | 11 | 8 | 18 | 14 |
| 25 | 2277528 | 2278069 | 0.03358 | 9 | 6 | 46 | 62 | 38 | 67 |
| 13 | 67058923 | 67059820 | 0.03496 | 2 | 1 | 20 | 7 | 13 | 16 |
| 19 | 51695613 | 51696050 | 0.03583 | 3 | 2 | 11 | 22 | 35 | 40 |
| 3 | 14659413 | 14660002 | 0.03583 | 2 | 0 | 10 | 16 | 12 | 12 |
| 23 | 39398709 | 39399704 | 0.03583 | 29 | 7 | 52 | 22 | 2 | 0 |
| 27 | 32713208 | 32714500 | 0.0367 | 16 | 0 | 22 | 33 | 41 | 28 |
| 4 | 117943112 | 117944284 | 0.03722 | 0 | 0 | 6 | 8 | 11 | 8 |
| 27 | 39771112 | 39772027 | 0.03722 | 10 | 36 | 0 | 0 | 15 | 4 |
| 15 | 29296418 | 29297309 | 0.03767 | 5 | 0 | 22 | 10 | 18 | 29 |
| 11 | 105593188 | 105593471 | 0.03802 | 2 | 0 | 7 | 15 | 19 | 15 |
| 18 | 54801139 | 54801524 | 0.03866 | 2 | 1 | 21 | 18 | 18 | 18 |
| 26 | 19337374 | 19337955 | 0.03866 | 2 | 3 | 18 | 18 | 20 | 18 |
| 11 | 68292468 | 68293471 | 0.03866 | 8 | 4 | 39 | 34 | 54 | 34 |
| 27 | 38114577 | 38115482 | 0.03866 | 8 | 2 | 35 | 26 | 35 | 32 |
| 23 | 28832819 | 28833352 | 0.03866 | 20 | 21 | 0 | 0 | 9 | 5 |
| 11 | 44457963 | 44458283 | 0.03866 | 13 | 2 | 24 | 57 | 38 | 53 |
| 21 | 35170189 | 35171105 | 0.03868 | 5 | 3 | 28 | 23 | 19 | 22 |
| 17 | 42833236 | 42834525 | 0.03868 | 15 | 21 | 0 | 3 | 0 | 0 |
| 12 | 31833814 | 31834660 | 0.03935 | 3 | 0 | 16 | 13 | 14 | 8 |
| 14 | 79188908 | 79190350 | 0.03951 | 129 | 279 | 175 | 0 | 167 | 131 |
| 18 | 62066012 | 62066482 | 0.03969 | 9 | 1 | 30 | 30 | 34 | 46 |
| 10 | 60335042 | 60335543 | 0.03984 | 20 | 38 | 2 | 0 | 16 | 10 |
| 19 | 42465712 | 42466121 | 0.03984 | 4 | 0 | 7 | 11 | 29 | 26 |
| 11 | 89100970 | 89101287 | 0.04186 | 2 | 1 | 16 | 10 | 7 | 16 |
| 7 | 79064603 | 79065454 | 0.04186 | 26 | 66 | 2 | 2 | 13 | 28 |
| 22 | 61320598 | 61321164 | 0.042 | 7 | 2 | 18 | 26 | 36 | 32 |
| 4 | 97777446 | 97777874 | 0.04241 | 3 | 2 | 25 | 10 | 15 | 28 |
| X | 124237017 | 124237416 | 0.04268 | 14 | 0 | 20 | 31 | 28 | 30 |
| X | 32323654 | 32324369 | 0.04414 | 0 | 1 | 10 | 12 | 8 | 20 |
| 25 | 29853719 | 29854177 | 0.04414 | 0 | 1 | 10 | 15 | 10 | 14 |
| 28 | 43433907 | 43434381 | 0.04414 | 2 | 0 | 11 | 12 | 14 | 9 |
| 27 | 17553193 | 17554805 | 0.04432 | 16 | 15 | 1 | 0 | 0 | 0 |
| 20 | 69861221 | 69861536 | 0.04436 | 6 | 2 | 23 | 28 | 29 | 27 |
| 25 | 36516804 | 36517131 | 0.04464 | 4 | 0 | 20 | 12 | 14 | 16 |
| 19 | 59762962 | 59763363 | 0.04479 | 9 | 20 | 0 | 0 | 0 | 0 |
| 23 | 48167410 | 48168916 | 0.04479 | 17 | 3 | 30 | 32 | 62 | 68 |
| 25 | 25255074 | 25255382 | 0.04479 | 1 | 0 | 13 | 10 | 8 | 21 |
| 4 | 8212970 | 8213369 | 0.04487 | 2 | 0 | 9 | 15 | 23 | 7 |
| 13 | 59070209 | 59070544 | 0.04487 | 0 | 0 | 3 | 7 | 7 | 12 |
| 9 | 72417137 | 72417500 | 0.04487 | 0 | 0 | 10 | 5 | 10 | 8 |
| 16 | 55213165 | 55213776 | 0.04568 | 30 | 26 | 0 | 7 | 4 | 0 |
| 14 | 3792831 | 3794274 | 0.04568 | 13 | 2 | 40 | 29 | 31 | 51 |
| 5 | 25933339 | 25934077 | 0.04613 | 5 | 1 | 25 | 20 | 22 | 43 |
| 3 | 56369048 | 56370180 | 0.04634 | 53 | 55 | 6 | 3 | 4 | 4 |
| 3 | 93284266 | 93285000 | 0.04634 | 20 | 0 | 29 | 24 | 35 | 47 |
| 11 | 92599560 | 92599989 | 0.04702 | 3 | 0 | 10 | 23 | 14 | 17 |
| 19 | 21078714 | 21079517 | 0.04702 | 2 | 0 | 6 | 26 | 24 | 4 |
| 7 | 16159081 | 16159903 | 0.04759 | 0 | 0 | 6 | 5 | 13 | 7 |
| 15 | 47395235 | 47395550 | 0.04759 | 0 | 0 | 6 | 5 | 14 | 6 |
| 19 | 59250927 | 59251945 | 0.04759 | 0 | 0 | 6 | 5 | 14 | 6 |
| 20 | 71489528 | 71489832 | 0.04759 | 2 | 3 | 21 | 22 | 27 | 23 |
| 15 | 53894557 | 53894894 | 0.04759 | 2 | 1 | 9 | 16 | 8 | 14 |
| 3 | 102949455 | 102950253 | 0.04759 | 27 | 5 | 63 | 100 | 77 | 89 |
| 7 | 47696146 | 47697493 | 0.04759 | 18 | 2 | 28 | 48 | 54 | 52 |
| 25 | 442569 | 443244 | 0.04759 | 0 | 6 | 28 | 12 | 18 | 16 |
| 18 | 44024776 | 44025416 | 0.04759 | 1 | 0 | 10 | 15 | 8 | 16 |
| 10 | 45236504 | 45236982 | 0.04759 | 3 | 0 | 8 | 16 | 14 | 10 |
| 1 | 145821684 | 145822254 | 0.04759 | 1 | 0 | 6 | 16 | 18 | 10 |
| 23 | 33408824 | 33409856 | 0.04759 | 14 | 18 | 0 | 2 | 0 | 0 |
| 5 | 32475060 | 32475427 | 0.04759 | 0 | 0 | 2 | 12 | 8 | 12 |
| 3 | 25026563 | 25026843 | 0.04893 | 894 | 219 | 2548 | 2666 | 2377 | 3987 |
| 22 | 50568940 | 50571578 | 0.04893 | 23 | 4 | 63 | 54 | 53 | 78 |
| 25 | 4024537 | 4024989 | 0.04893 | 13 | 1 | 16 | 25 | 23 | 38 |
| 3 | 25026073 | 25026351 | 0.04912 | 891 | 219 | 2535 | 2658 | 2371 | 3969 |
| 13 | 40595502 | 40596317 | 0.04956 | 2 | 0 | 18 | 10 | 16 | 5 |
| 28 | 27376723 | 27377660 | 0.04956 | 0 | 0 | 4 | 13 | 7 | 5 |

^1^Aligned to: UMD_3.1 (<http://www.ncbi.nlm.nih.gov/assembly/GCA_000003055.4>)
