# Supplementary material for: DNA methylation patterns in peripheral blood mononuclear cells from Holstein cattle with variable milk yield
Source: BMC Genomics. 2018 Oct 11;19:744. doi: 10.1186/s12864-018-5124-9 (PMC6182825; doi:10.1186/s12864-018-5124-9)
Supplement: Supplementary file 11 — Figure S2. The location and surrounding genomic region for the top 11 most significant case-control differentially methylated regions. NCBI Genome Data Viewer of regions that harbor putative DMR with the location of each DMR and identification of nearby genes from NCBI Bos taurus Annotation Release 105, 2016-01-26. (PDF 294 kb) [file 12864_2018_5124_MOESM11_ESM.pdf]

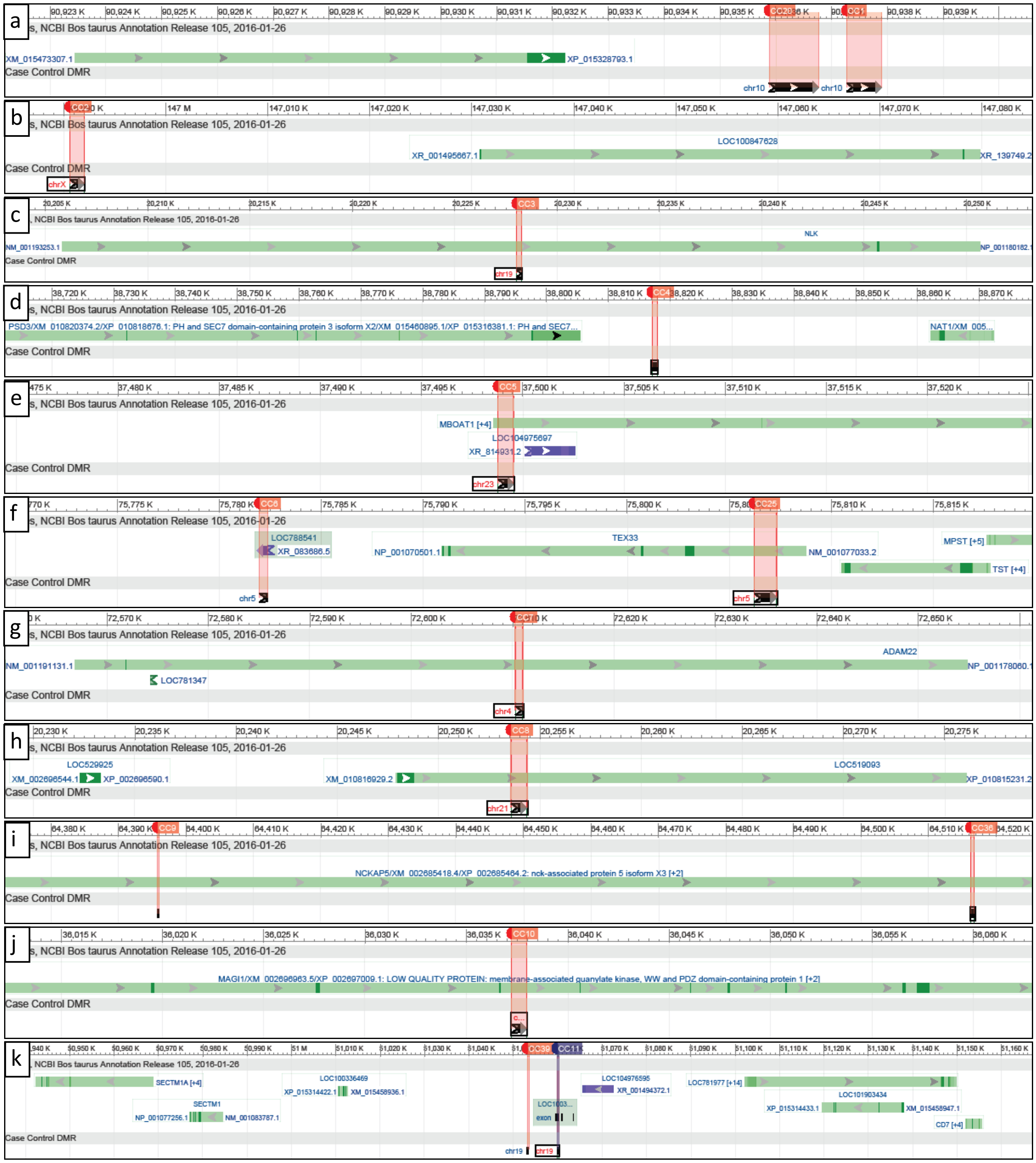


Figure S2. The location and surrounding genomic region for the top 11 most significant case-control differentially methylated regions; a. *LOC107133457* = neurexin-3-like protein coding gene; b. *LOC100847628* is a non-characterized ncRNA; c. *NLK* = nemo like kinase; d. *PSD3* = pleckstrin and Sec7 domain containing 3; e. *MBOAT1* = membrane bound O-acyltransferase domain containing 1; f. *LOC788541* = 60S ribosomal protein L7 pseudogene and *TEX33* = testis expressed 33; g. *ADAM22* = ADAM metallopeptidase domain 22; h. *LOC519093* = myeloid-associated differentiation marker-like protein coding gene; i. *NCKAP5* = NCK associated protein 5; j. *MAGI1* = membrane associated guanylate kinase, WW and PDZ domain containing 1; k. *LOC100300790* = secreted and transmembrane protein 1A-like pseudogene.
