# Supplementary material for: DNA methylation patterns in peripheral blood mononuclear cells from Holstein cattle with variable milk yield
Source: BMC Genomics. 2018 Oct 11;19:744. doi: 10.1186/s12864-018-5124-9 (PMC6182825; doi:10.1186/s12864-018-5124-9)
Supplement: Supplementary file 12 — Figure S3. The location and surrounding genomic region for the top 10 most significant environmental differentially methylated regions. NCBI Genome Data Viewer of regions that harbor putative DMR with the location of each DMR and identification of nearby genes from NCBI Bos taurus Annotation Release 105, 2016-01-26. (PDF 119 kb) [file 12864_2018_5124_MOESM12_ESM.pdf]

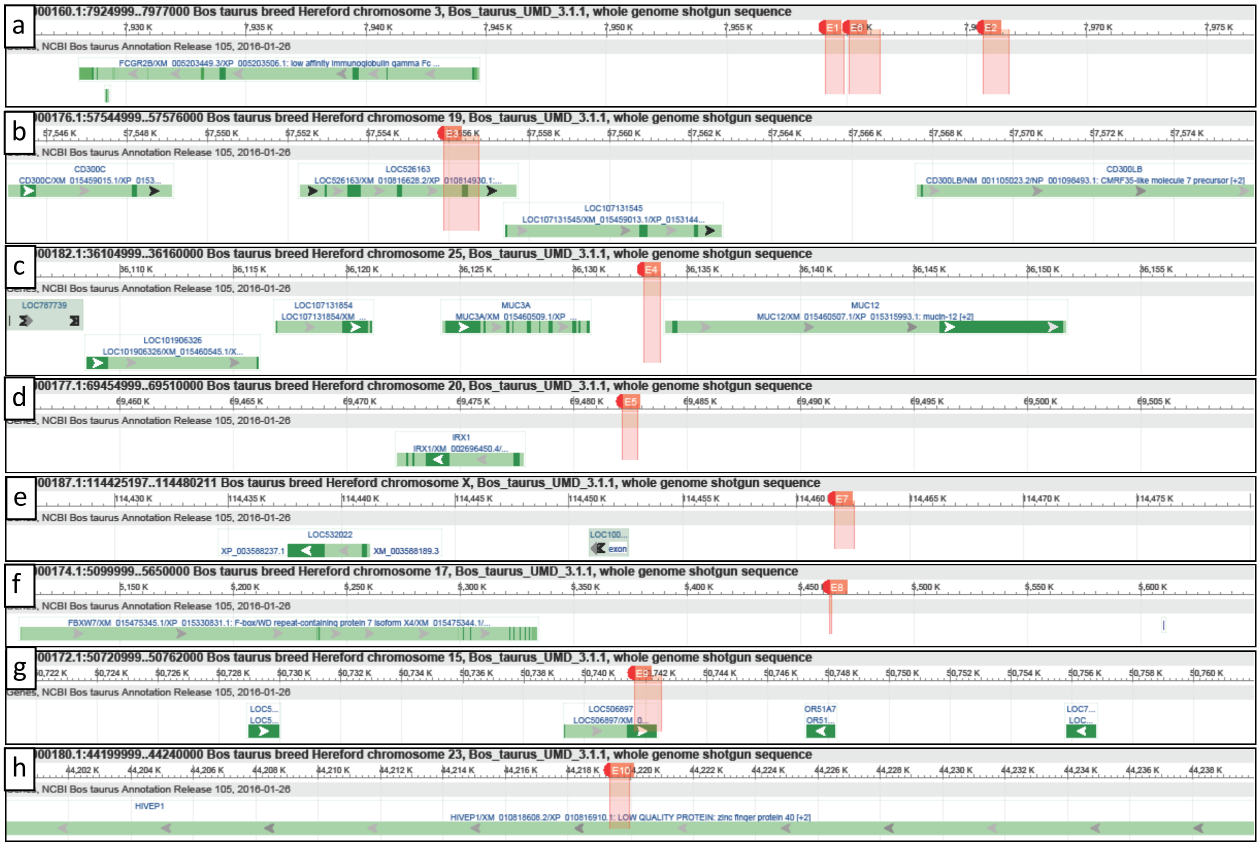

Figure S3. The location and surrounding genomic region for the top 10 most significant environmental differentially methylated regions; a. *FCGR2B* = Fc fragment of IgG receptor IIb; b. *LOC526163* = CMRF35-like molecule 6 protein coding gene; c. *MUC12* = mucin 12; d. *IRX1* = Iroquois homeobox; e. *LOC100847359* = serine/arginine-rich splicing factor 2 pseudogene; f. *FBXW7* = F-box/WD repeat-containing protein 7 isoform; g. *LOC506897* = olfactory receptor 51G2; h. *HIVEP1* = human immunodeficiency virus type I enhancer binding protein 1.
