# Supplementary material for: DNA methylation patterns in peripheral blood mononuclear cells from Holstein cattle with variable milk yield
Source: BMC Genomics. 2018 Oct 11;19:744. doi: 10.1186/s12864-018-5124-9 (PMC6182825; doi:10.1186/s12864-018-5124-9)
Supplement: Supplementary file 13 — Table S10. Eight homeobox genes associated with a differentially methylated region (DMR) that were part of a single functional annotation chart with an FDR adjusted P < 0.05. Ensembl gene ID, gene name, location of DMR, and location of gene TSS. (DOCX 12 kb) [file 12864_2018_5124_MOESM13_ESM.docx]

Table S10. Eight homeobox genes associated with a differentially methylated region (DMR) that were part of a single functional annotation chart with an FDR adjusted *P<*0.05.

| Ensembl Gene ID | Gene Name | chromosome | transcription start site | DMR start site | DMR end site |
| --- | --- | --- | --- | --- | --- |
| ENSBTAG00000005938 | BarH like homeobox 2 (BARHL2) | 3 | 52,691,875 | 52,689,106 | 52,690,229 |
| ENSBTAG00000005525 | LIM homeobox 6 (LHX6) | 11 | 93,090,242 | 93,086,653 | 93,086,977 |
| ENSBTAG00000012025 | LIM homeobox transcription factor 1 alpha (LMX1A) | 3 | 3,692,342 | 3,692,188 | 3,694,830 |
| ENSBTAG00000010277 | NK2 homeobox 2 (NKX2-2) | 13 | 41,107,079 | 41,103,824 | 41,104,166 |
| ENSBTAG00000020568 | NK2 homeobox 5 (NKX2-5) | 20 | 4,821,534 | 4,819,352 | 4,821,155 |
| ENSBTAG00000005741 | distal-less homeobox 2 (DLX2) | 2 | 24,489,073 | 24,490,163 | 24,490,861 |
| ENSBTAG00000000809 | iroquois homeobox 1 (IRX1) | 20 | 69,477,632 | 69,482,161 | 69,482,790 |
| ENSBTAG00000003335 | visual system homeobox 1 (VSX1) | 13 | 43,110,540 | 43,112,930 | 43,113,484 |
