# Supplementary material for: DNA methylation patterns in peripheral blood mononuclear cells from Holstein cattle with variable milk yield
Source: BMC Genomics. 2018 Oct 11;19:744. doi: 10.1186/s12864-018-5124-9 (PMC6182825; doi:10.1186/s12864-018-5124-9)
Supplement: Supplementary file 14 — Table S11. 48 sampling scenarios implemented in permutations to identify significance thresholds for identifying differentially methylated regions. A list of the high and low cow selected for each herd for each of the 48 sampling strategies. (DOCX 13 kb) [file 12864_2018_5124_MOESM14_ESM.docx]

Table S11. 48 sampling scenarios implemented in permutations to identify significance thresholds for identifying differentially methylated regions.

| Sample  Scenario^1^ | Herd environment 1 | |  | Herd environment 2 | |  | Herd environment 3 | |
| --- | --- | --- | --- | --- | --- | --- | --- | --- |
|  | High | Low |  | High | Low |  | Cow 1 | Cow 2 |
| 1 | F1H | F1L |  | F2H | F2L |  | F3I | F4I |
| 2 | F1H | F1L |  | F2H | F2L |  | F4I | F3I |
| 3 | F1H | F1L |  | F2L | F2H |  | F3I | F4I |
| 4 | F1H | F1L |  | F2L | F2H |  | F4I | F3I |
| 5 | F1H | F1L |  | F3I | F4I |  | F2H | F2L |
| 6 | F1H | F1L |  | F4I | F3I |  | F2H | F2L |
| 7 | F1H | F1L |  | F3I | F4I |  | F2L | F2H |
| 8 | F1H | F1L |  | F4I | F3I |  | F2L | F2H |
| 9 | F1L | F1H |  | F2H | F2L |  | F3I | F4I |
| 10 | F1L | F1H |  | F2H | F2L |  | F4I | F3I |
| 11 | F1L | F1H |  | F2L | F2H |  | F3I | F4I |
| 12 | F1L | F1H |  | F2L | F2H |  | F4I | F3I |
| 13 | F1L | F1H |  | F3I | F4I |  | F2H | F2L |
| 14 | F1L | F1H |  | F4I | F3I |  | F2H | F2L |
| 15 | F1L | F1H |  | F3I | F4I |  | F2L | F2H |
| 16 | F1L | F1H |  | F4I | F3I |  | F2L | F2H |
| 17 | F2H | F2L |  | F1H | F1L |  | F3I | F4I |
| 18 | F2H | F2L |  | F1H | F1L |  | F4I | F3I |
| 19 | F2H | F2L |  | F1L | F1H |  | F3I | F4I |
| 20 | F2H | F2L |  | F1L | F1H |  | F4I | F3I |
| 21 | F2H | F2L |  | F3I | F4I |  | F1H | F1L |
| 22 | F2H | F2L |  | F4I | F3I |  | F1H | F1L |
| 23 | F2H | F2L |  | F3I | F4I |  | F1L | F1H |
| 24 | F2H | F2L |  | F4I | F3I |  | F1L | F1H |
| 25 | F2L | F2H |  | F1H | F1L |  | F3I | F4I |
| 26 | F2L | F2H |  | F1H | F1L |  | F4I | F3I |
| 27 | F2L | F2H |  | F1L | F1H |  | F3I | F4I |
| 28 | F2L | F2H |  | F1L | F1H |  | F4I | F3I |
| 29 | F2L | F2H |  | F3I | F4I |  | F1H | F1L |
| 30 | F2L | F2H |  | F4I | F3I |  | F1H | F1L |
| 31 | F2L | F2H |  | F3I | F4I |  | F1L | F1H |
| 32 | F2L | F2H |  | F4I | F3I |  | F1L | F1H |
| 33 | F3I | F4I |  | F1H | F1L |  | F2H | F2L |
| 34 | F3I | F4I |  | F1H | F1L |  | F2L | F2H |
| 35 | F3I | F4I |  | F1L | F1H |  | F2H | F2L |
| 36 | F3I | F4I |  | F1L | F1H |  | F2L | F2H |
| 37 | F3I | F4I |  | F2H | F2L |  | F1H | F1L |
| 38 | F3I | F4I |  | F2L | F2H |  | F1H | F1L |
| 39 | F3I | F4I |  | F2H | F2L |  | F1L | F1H |
| 40 | F3I | F4I |  | F2L | F2H |  | F1L | F1H |
| 41 | F4I | F3I |  | F1H | F1L |  | F2H | F2L |
| 42 | F4I | F3I |  | F1H | F1L |  | F2L | F2H |
| 43 | F4I | F3I |  | F1L | F1H |  | F2H | F2L |
| 44 | F4I | F3I |  | F1L | F1H |  | F2L | F2H |
| 45 | F4I | F3I |  | F2H | F2L |  | F1H | F1L |
| 46 | F4I | F3I |  | F2L | F2H |  | F1H | F1L |
| 47 | F4I | F3I |  | F2H | F2L |  | F1L | F1H |
| 48 | F4I | F3I |  | F2L | F2H |  | F1L | F1H |

^1^Cows identified in Table 1: F1H=farm 1 high; F1L=farm 1 low; F2H=farm 2 high; F2L=farm 2 low; F3I=farm 3 intermediate; F4I=farm 4 intermediate.
